# Type 2 Diabetes Risk Alleles Demonstrate Extreme Directional Differentiation among Human Populations, Compared to Other Diseases

**DOI:** 10.1371/journal.pgen.1002621

**Published:** 2012-04-12

**Authors:** Rong Chen, Erik Corona, Martin Sikora, Joel T. Dudley, Alex A. Morgan, Andres Moreno-Estrada, Geoffrey B. Nilsen, David Ruau, Stephen E. Lincoln, Carlos D. Bustamante, Atul J. Butte

**Affiliations:** 1 Division of Systems Medicine, Department of Pediatrics, Stanford University School of Medicine, Stanford, California, United States of America; 2 Lucile Packard Children's Hospital, Palo Alto, California, United States of America; 3 Program in Biomedical Informatics, Stanford University School of Medicine, Stanford, California, United States of America; 4 Department of Genetics, Stanford University School of Medicine, Stanford, California, United States of America; 5 Complete Genomics, Mountain View, California, United States of America; Vanderbilt University School of Medicine, United States of America

## Abstract

Many disease-susceptible SNPs exhibit significant disparity in ancestral and derived allele frequencies across worldwide populations. While previous studies have examined population differentiation of alleles at specific SNPs, global ethnic patterns of ensembles of disease risk alleles across human diseases are unexamined. To examine these patterns, we manually curated ethnic disease association data from 5,065 papers on human genetic studies representing 1,495 diseases, recording the precise risk alleles and their measured population frequencies and estimated effect sizes. We systematically compared the population frequencies of cross-ethnic risk alleles for each disease across 1,397 individuals from 11 HapMap populations, 1,064 individuals from 53 HGDP populations, and 49 individuals with whole-genome sequences from 10 populations. Type 2 diabetes (T2D) demonstrated extreme directional differentiation of risk allele frequencies across human populations, compared with null distributions of European-frequency matched control genomic alleles and risk alleles for other diseases. Most T2D risk alleles share a consistent pattern of decreasing frequencies along human migration into East Asia. Furthermore, we show that these patterns contribute to disparities in predicted genetic risk across 1,397 HapMap individuals, T2D genetic risk being consistently higher for individuals in the African populations and lower in the Asian populations, irrespective of the ethnicity considered in the initial discovery of risk alleles. We observed a similar pattern in the distribution of T2D Genetic Risk Scores, which are associated with an increased risk of developing diabetes in the Diabetes Prevention Program cohort, for the same individuals. This disparity may be attributable to the promotion of energy storage and usage appropriate to environments and inconsistent energy intake. Our results indicate that the differential frequencies of T2D risk alleles may contribute to the observed disparity in T2D incidence rates across ethnic populations.

## Author Summary

We identified 12 risk alleles that had been validated to increase the risk of type 2 diabetes (T2D) in five or more different subpopulations. These risk alleles share a consistent pattern of decreasing frequencies in the human genomes from Sub-Saharan Africa and through Europe to East Asia regions. These differential frequencies are statistically significant, compared with European frequency-matched control genomic alleles and risk alleles for other diseases. The differential frequencies of T2D risk alleles further caused the significant differentiation of genetic risks of T2D, with higher risk in the African and lower risk in the Asian populations. The differences might be caused by the promotion of energy storage and usage appropriate to environments and inconsistent energy intake. Future evolutionary and environmental analysis of this unique pattern may provide further insight on the origin of T2D and modern disparities in T2D incidence.

## Introduction

The global rise in incidence of type 2 diabetes (T2D) has been called a pandemic, and is now commonly identified as a major international health concern [1]. Although environmental factors play a substantial role in the etiology of T2D, genetic susceptibility has been established as a key component of risk [2], [3]. However, the potential disparities in genetic risks for T2D and other major diseases across different ethnic groups and subpopulations are poorly characterized. In this study, we evaluate the hypothesis that disparities in global patterns of T2D risk allele frequencies contribute to disparities in genetic risk of T2D across diverse global populations, compared with genomic background and other diseases.

We evaluate this hypothesis using three catalogs of genetic variation sampled from diverse ethnic populations [4], [5] and published associations between genetic variants and disease traits [6]. Specifically, we include 1,397 individuals from 11 populations represented in the HapMap project [4] and to follow-up findings, we added 1,064 individuals from 53 indigenous populations across the world in the Human Genome Diversity Panel (HGDP) [7]. Additionally, we recently sequenced and publically released the whole genomes of 49 individuals from 10 diverse populations. Disease associated variants were collated from the thousands of genetic variants associated with increased disease risk through thousands of genome-wide and candidate association studies [8], leveraging our previous efforts to catalog, summarize, and integrate these risk variants across hundreds of human diseases [6].

Enabled by the catalogs described above, a recent analysis demonstrated wide variations in the frequencies of some disease-susceptible risk alleles across 11 HapMap subpopulations [9], but with the caveat that many of the well-known disease SNPs were discovered in population of European ancestry [10]. To further explore the association between individual genetic risks in subpopulations, we propose conducting a systematic evaluation of the differentiation of the risk allele frequencies (RAFs) and individual genetic risks across population groups. The results aim to further elucidate the role of ethnicity in clinical diagnosis of genetic disease risk and also provide guidance towards discovering additional disease-susceptible genetic variants from diverse population groups.

We are focusing on T2D and its comparison with other diseases because of its pandemic prevalence and a striking feature of extreme population differentiation we find in this study. Inconsistent results have been previously reported on the population differentiation of RAF and genetic risk of T2D [11]–[15]. However, to our knowledge, most of the studies have been focused on individual SNPs, and none of them considered the consistency of directions of population differentiation across all T2D-susceptible risk alleles. Furthermore, none of these previous studies have systematically compared the population differentiation between T2D and other diseases.

In this study, we systematically calculate the directional population differentiation of RAFs across cross-ethnic T2D risk alleles using genotyping data from diverse population groups in both HapMap and HGDP, and evaluate the statistical significance of differentiation against genomic background and other diseases. We then calculate the predicted genetic risk (PGR) of 1,397 individuals from 11 HapMap populations, and identify diseases with significant differentiation across diverse population groups.

## Results

### Each of 12 cross-ethnic T2D SNPs share the same risk allele and similar odds ratios in 34 studied populations

We manually curated thousands of papers covering human disease genetics and identified independent risk alleles increasing the risk of Type 2 diabetes (T2D) across diverse population groups using the following procedure. Specifically, we curated 50,730 SNPs associating with 1,495 human diseases from 5,065 papers, and built a quantitative human disease-SNP association database, called VARiant-INforming MEDicine (Varimed) [6]. Of these, 8,377 SNPs for 437 diseases had been initially reported with a p-value<10^−6^, and only these SNPs were used in this study. From 120 T2D SNPs reported in 295 studies in 132 papers with p-value<10^−6^, we identified cross-ethnic SNPs as being replicated in five or more different populations. We then removed SNPs that are in linkage disequilibrium (R^2^>0.7) in Caucasian population in HapMap (merged HapMap 2+3) [4]. Unless otherwise specified, HapMap were referred to this merged version throughout this study. In total, we identified 12 independent cross-ethnic risk alleles increasing T2D risk, including one in *SLC30A8*, *IGF2BP2*, *KCNJ11*, *FTO*, and two in *TCF7L2* (R^2^ = 0.512), two in *CDKAL1* (R^2^ = 0.677), two in *KCNQ1* (R^2^ = 0.425), and two outside gene regions. All 12 SNPs had been validated to associate with T2D with p<5×10^−8^ in two or more diverse populations, except rs11196205 (Table S1). Previous independent reviews and meta-analysis also support the notion that these 12 SNPs are likely to be generally relevant to T2D across populations [16]–[19].

For each of these 12 SNPs, we found that the same allele had been consistently identified as the risk allele with similar odds ratios across 34 studied populations (Figure S1, Table S2), though three SNPs show high heterogeneity of allelic odds ratios between studies (I^2^>75%) and three SNPs show moderate heterogeneity (I^2^>50%) (Table S3). To evaluate whether the shared risk allele and effect sizes were caused by the bias towards cross-ethnic SNPs, we identified a second set of replicated T2D SNPs as those that were replicated in two independent papers with p<5×10^−8^ without the requirement of five or more studied populations. We identified 11 independent replicated T2D SNPs, including 10 cross-ethnic SNPs and rs864745 in *JAZF1*. Each of these 11 replicated T2D SNPs shared the same risk allele and similar effect sizes across all studied populations, suggesting that they are the best representatives of the causal alleles based on the current data.

### T2D risk alleles share a similar pattern of frequency distribution across 11 HapMap populations

**Figure 1:**
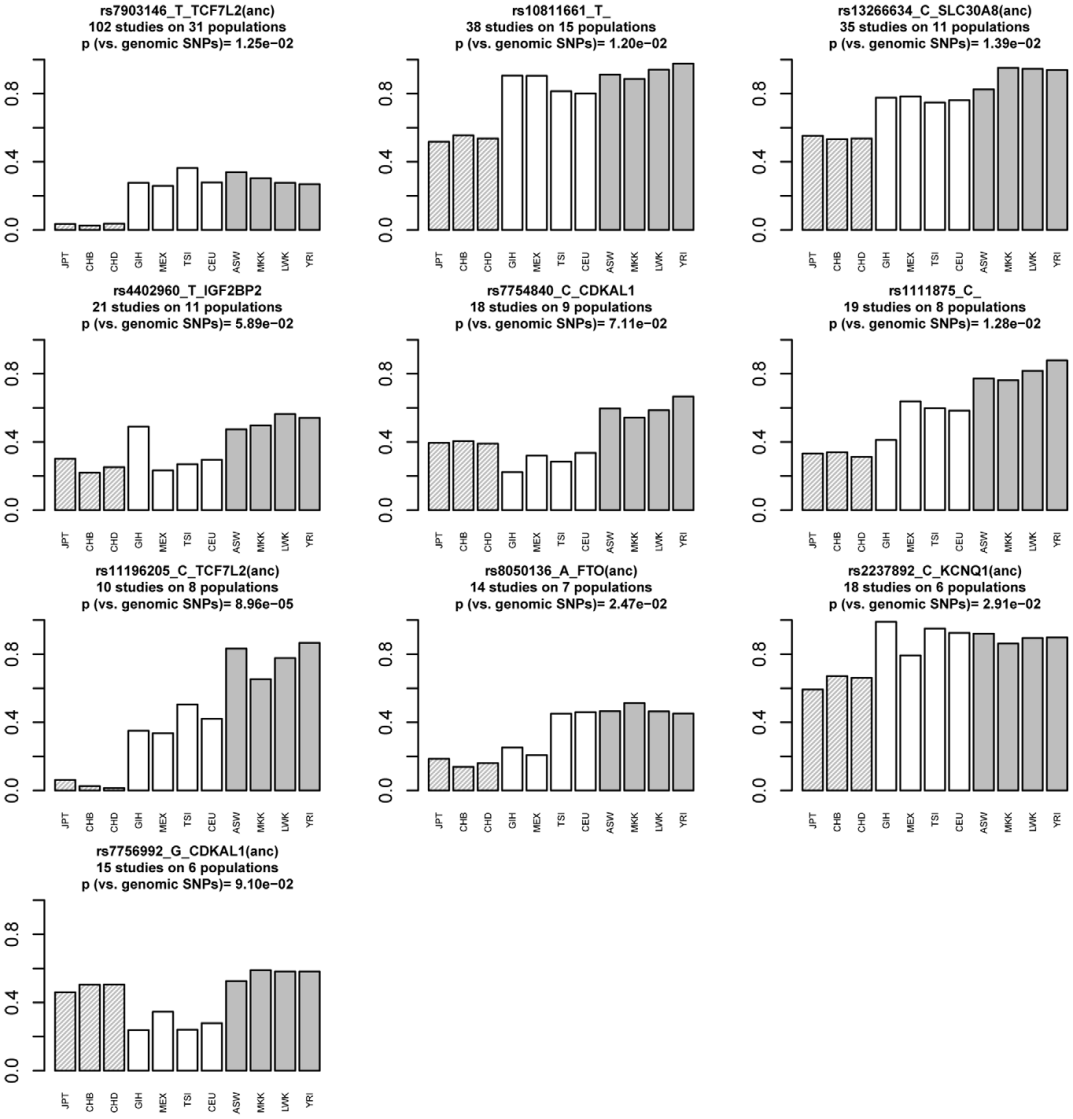
T2D risk alleles share a similar pattern of frequencies across 11 HapMap populations with higher frequencies in African and lower frequencies in Asian populations. Risk-allele frequencies (RAF) were shown as heights of bars across the 11 HapMap populations at 10 independent cross-ethnic T2D SNPs, which had been replicated to associate with T2D in five or more diverse populations. Two cross-ethnic T2D SNPs including rs5219 and rs2074196 were not shown due to the lack of RAF data. Most T2D SNPs share the same pattern of population differentiation with higher RAFs in the African (ASW, MKK, LWK, YRI, colored in grey and listed on the right) and lower RAFs in the Asian populations (JPT, CHB, CHD, shaded and listed on the left), compared to the RAFs in the European populations. For each risk allele, a p value was calculated as the percentage of genomic alleles with matched frequencies in the European populations that show both higher African RAF and lower Asian RAF than the observed. For example, only 8.96×10^−5^ matched genomic alleles show both higher frequencies in the African populations and lower frequencies in the Asian populations than the observed frequencies of rs11196205. All T2D risk alleles except for two alleles in *CDKAL1* show statistically significantly larger population differentiation than European frequency-matched genome alleles (p<0.05). The (anc) in the sub-title indicates that the risk allele is the ancestral allele according to mammalian sequence data, retrieved from dbSNP.

We then plotted the RAF across 11 HapMap populations at independent cross-ethnic T2D SNPs and evaluated their significance of population differentiation against frequency-matched control genomic alleles. Two risk alleles, including rs5219 in *KCNJ11* and rs2074196 in *KCNQ1*, were not analyzed due to the lack of frequency data. Most of remaining ten cross-ethnic T2D risk alleles shared a similar pattern showing the highest RAF in African populations and the lowest RAF in Asian populations (Figure 1). To evaluate the significance of differentiation, we retrieved all genomic alleles that shared the same European frequencies and calculated how many of those had both higher African frequencies and lower Asian frequencies than what we observed at each risk allele. Seven out of ten SNPs showed larger differentiation of RAF than 95% European frequency-matched control genomic alleles (Figure 1). Interestingly, none of the protective alleles of these ten SNPs exhibited significantly higher frequencies in the African population groups.

### Decreasing RAF from Sub-Saharan Africa to East Asia regions at cross-ethnic T2D SNPs in HGDP individuals

**Figure 2:**
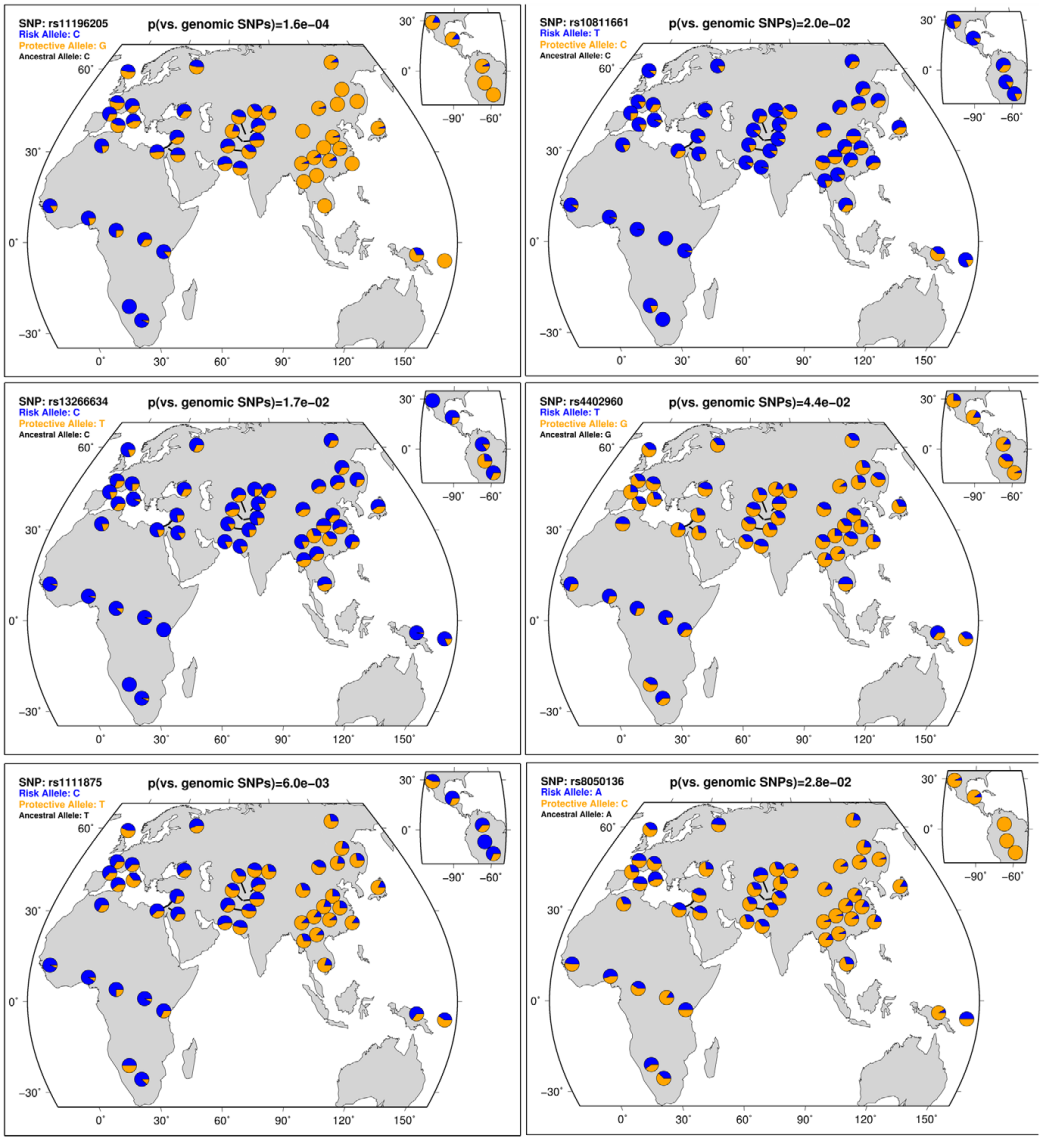
HGDP data show that T2D risk allele frequencies decrease from Sub-Saharan Africa through Europe to East Asia. The frequencies of six cross-ethnic T2D risk alleles were shown as dark blue wedges across the 53 populations, calculated from 1,064 individuals in the Human Genome Diversity Panel (HGDP) [7]. All T2D risk alleles decrease frequencies gradually from Sub-Saharan Africa through Europe to East Asia. A similar pattern was observed in the remaining five T2D risk alleles in Figure S2. The central map shows the Sub-Saharan Africa region on the lower left and the East Asia region on the right. The smaller map shows Mexico and South America regions. Similar to Figure 1, p values were calculated as the percentage of genomic alleles with matched frequencies in the European populations that show both higher frequencies in the Sub-Saharan Africa regions and lower frequencies in the East Asia regions than the observed RAF. All T2D SNPs except for two in *CDKAL1* show statistically significantly larger population differentiation than the frequency-matched genomic alleles (p<0.05). The (anc) in the sub-title indicates that the risk allele is the ancestral allele according to mammalian sequence data, retrieved from dbSNP.

We then calculated T2D RAF in 53 indigenous populations from 1,064 individuals in the Human Genome Diversity Panel (HGDP) [20]. One risk allele, rs5219 in *KCNJ11* was not analyzed due to the lack of frequency data. Nine out of the remaining 11 T2D SNPs exhibited larger differentiation of RAF than 95% European frequency-matched control genomic alleles, except for two SNPs in *CDKAL1* (Six SNPs in Figure 2, five SNPs in Figure S2). There is a clear pattern of gradually decreasing RAF from the Sub-Saharan Africa to the East Asia through the Europe regions, which was shared among all T2D SNPs.

### Higher *F*_ST_ values at T2D SNPs than frequency matched control genomic SNPs

To evaluate whether the observed allele frequency differences among continental groups for T2D SNPs are unusually high, we calculated *F*_ST_ as a measure of population differentiation [21]. Specifically, for each of ten cross-ethnic SNPs (rs5219 and rs2074196 were not analyzed due to the lack of frequency data), we calculated a global as well as three pairwise *F*_ST_ values by pooling populations for each of the three major geographic regions: Africa, Europe, and East Asia. We then compared them to the genome-wide distribution for allele frequency matched SNPs (defined as within the same 5% minor allele frequency bin in the pooled European samples). Consistent with previous results [12], [13], we found that all T2D SNPs showed elevated *F*_ST_ values, with five out of ten T2D SNPs among the top 10%, and one of them (rs11196205 in *TCF7L2*) among the top 1% of the empirical distribution for at least one of the four population comparisons (Figure S3). Overall, *F*_ST_ values were significantly higher in T2D SNPs than the frequency matched genomic SNPs in global *F*_ST_ (p = 0.0057), using a Mann-Whitney U test. Of the pairwise comparisons, *F*_ST_ of both African vs. East Asian (p = 0.0041) and East Asian vs. European (p = 0.025) were also highly significant, whereas African vs. European was not (p = 0.3). Thus, *F*_ST_ values also support our findings of extreme population differentiation at T2D SNPs.

### T2D risk alleles as an ensemble showed significantly larger differentiation than other diseases using a novel directional population differentiation method

**Figure 3:**
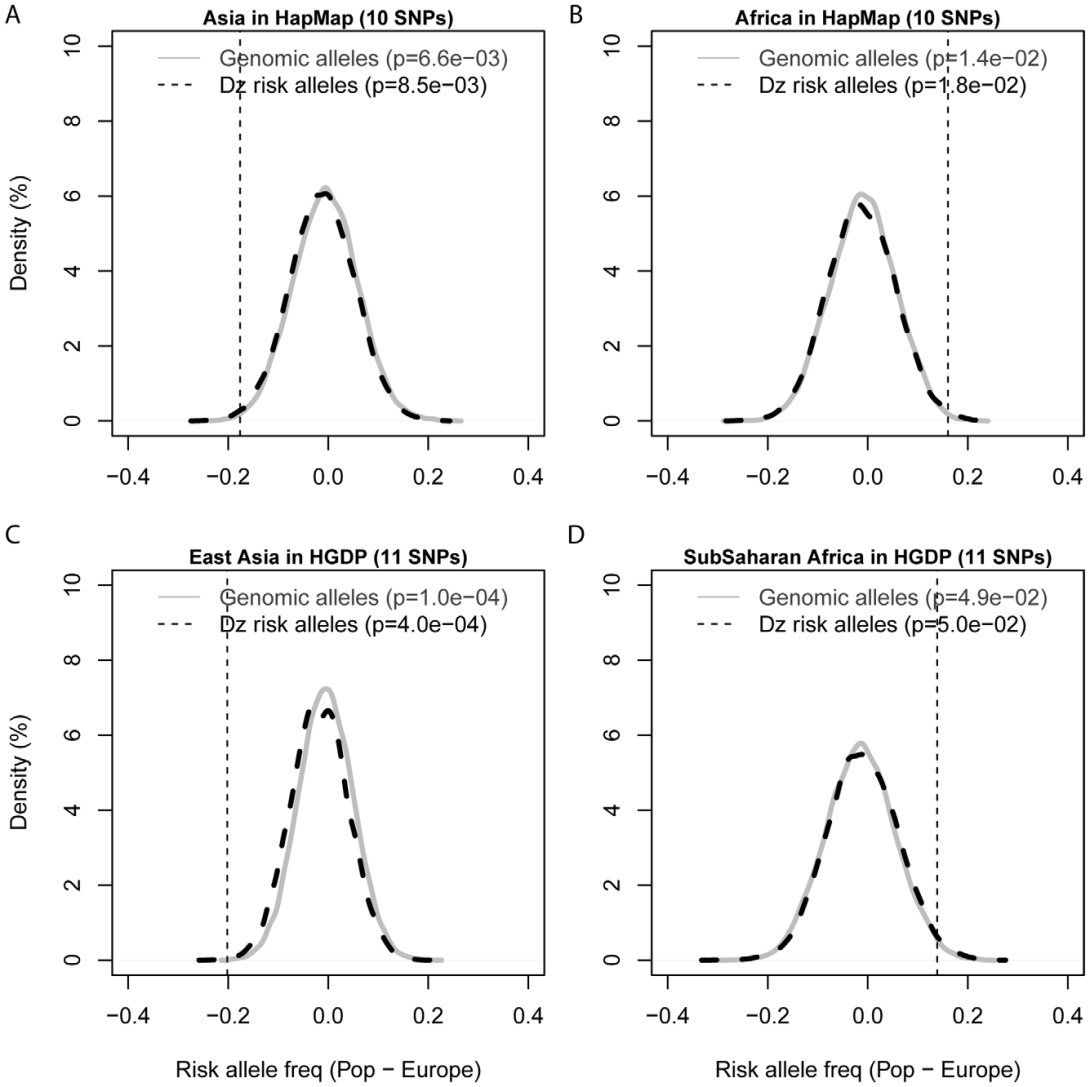
An ensemble of T2D risk alleles demonstrates significant directional differentiation of frequencies among human populations, compared to genomic control alleles and risk alleles for other diseases. Eleven independent cross-ethnic T2D risk alleles were combined as an ensemble to calculate the average increased frequencies in the populations in Asia (A) and Africa (B) from HapMap, and the East Asia (C) and Sub-Saharan Africa (D) from HGDP, compared with the frequencies in the European populations. The average increased RAFs of T2D risk alleles are shown as dotted vertical lines, and compared against the null distributions of average increased RAFs of 11 alleles randomly drawn from genomic alleles (solid black curve) and disease-susceptible risk alleles (dashed grey curve) that share the same allele frequencies with T2D risk alleles in the European populations. Two-side p values were calculated by comparing dotted vertical lines against the null distributions of frequency-matched control genomic alleles and risk alleles of other diseases. SNPs used in each figure were summarized in Table S4.

F*_ST_* has been widely used to evaluate population differentiation; however, it does not take into account whether risk alleles share the same direction of population differentiation. Here, we developed a novel directional population differentiation method by comparing the average increased frequencies across all T2D risk alleles against the null distribution of European frequency-matched control genomic alleles. We randomly drew 10 genomic alleles that share similar frequencies with the T2D risk alleles in the European populations. Then we calculated their average increased frequencies in the African compared with European populations, which is defined as RAF_African_−RAF_European_. Repeating the process 10,000 times, we got a null distribution of increased frequencies between African and European populations. T2D risk alleles as an ensemble demonstrated significantly higher RAF in the HapMap African populations against the null distribution of control genomic alleles with a two-side p value of 1.4×10^−2^ (Figure 3B). We adopted the two-side p value to make our method applicable to any new disease without any prior knowledge on whether RAF_African_−RAF_European_ is larger or smaller than 0. T2D risk alleles as an ensemble showed significantly higher RAF in the populations in the Sub-Saharan Africa regions (p = 4.9×10^−2^, Figure 3D), and significantly lower RAF in the East Asia regions (p = 1.0×10^−4^, Figure 3C), compared to control genomic alleles. We also observed very similar results when analyzing the directional population differentiation using a single T2D SNP from each gene.

We then evaluated whether this increased RAF in the Sub-Saharan Africa regions was significant against risk alleles from hundreds of other diseases. As a background, we extracted 15,649 risk alleles that had previously been reported as significantly associated with 975 human diseases from Varimed [6]. We randomly drew 11 risk alleles which share similar frequencies with T2D risk alleles in the European populations and calculated a null distribution of increased frequencies. We found that T2D risk alleles showed significantly higher RAF in the Sub-Saharan Africa region than risk alleles from other diseases (Figure 3D, p = 0.05). Similarly, we found that T2D risk alleles showed significantly lower RAF in the HapMap Asia (p = 8.5×10^−3^, Figure 3A) and HGDP East Asia regions (p = 4.0×10^−4^, Figure 3C). Therefore, our novel method demonstrated that T2D risk alleles as an ensemble showed significantly larger population differentiation than frequency-matched control genomic alleles and risk alleles from hundreds of other diseases.

**Figure 4:**
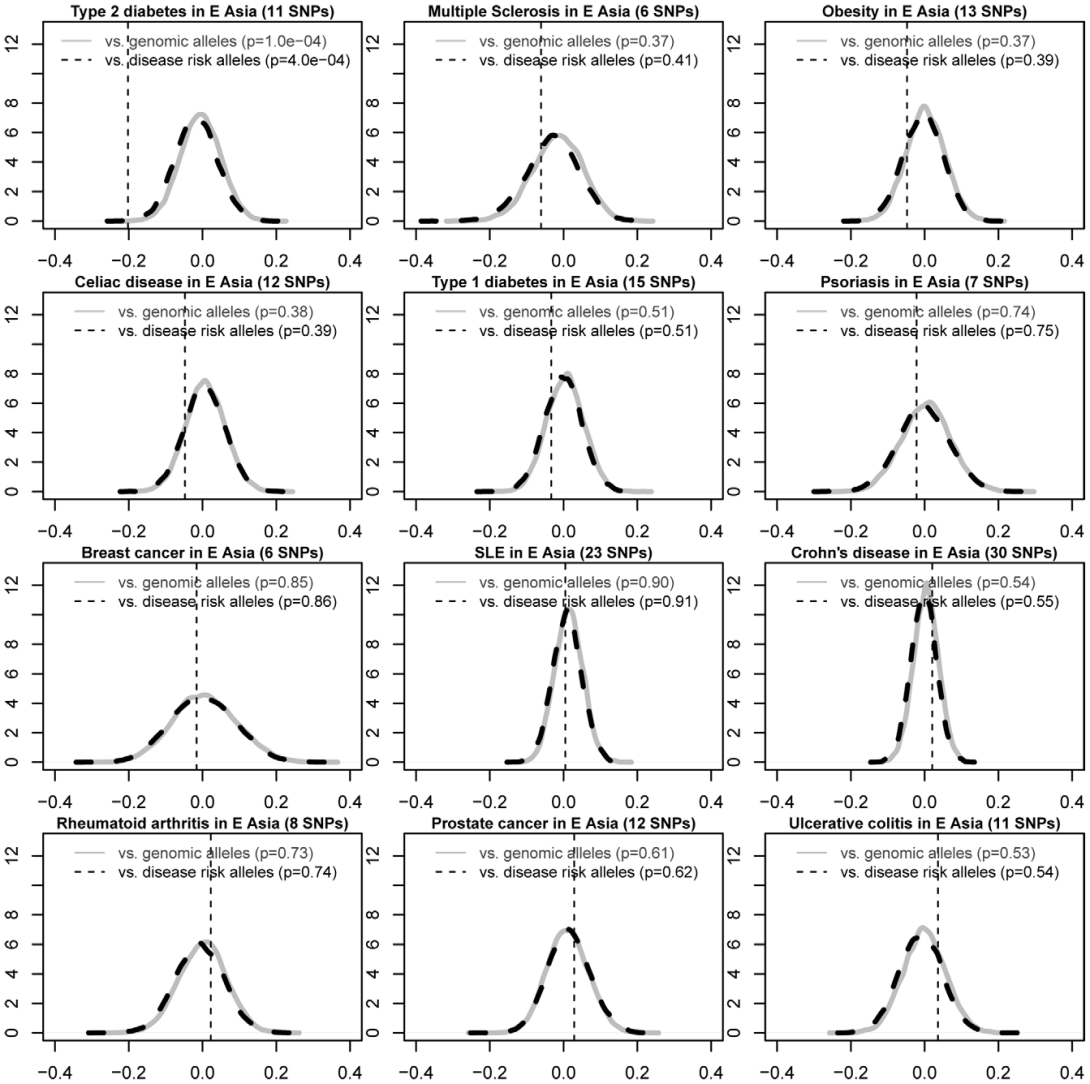
Differential risk allele frequencies in the East Asian populations for 12 common diseases, compared with the frequencies in the European. We identified 12 common diseases with five or more independent cross-ethnic risk alleles from Varimed. Similar with Figure 3, we plotted the average increased RAFs in the East Asia regions in the HGDP for each of 12 diseases, against the null distributions of frequency-matched control genomic alleles and risk alleles for other diseases. We ordered these 12 diseases by the increased RAF for a direct comparison. T2D was the only disease showing significantly lower RAFs in the East Asian populations. Two-side p values were calculated by comparing dotted vertical lines against the null distributions of frequency-matched control genomic alleles and risk alleles of other diseases. SNPs used in each figure were summarized in Table S4.

**Figure 5:**
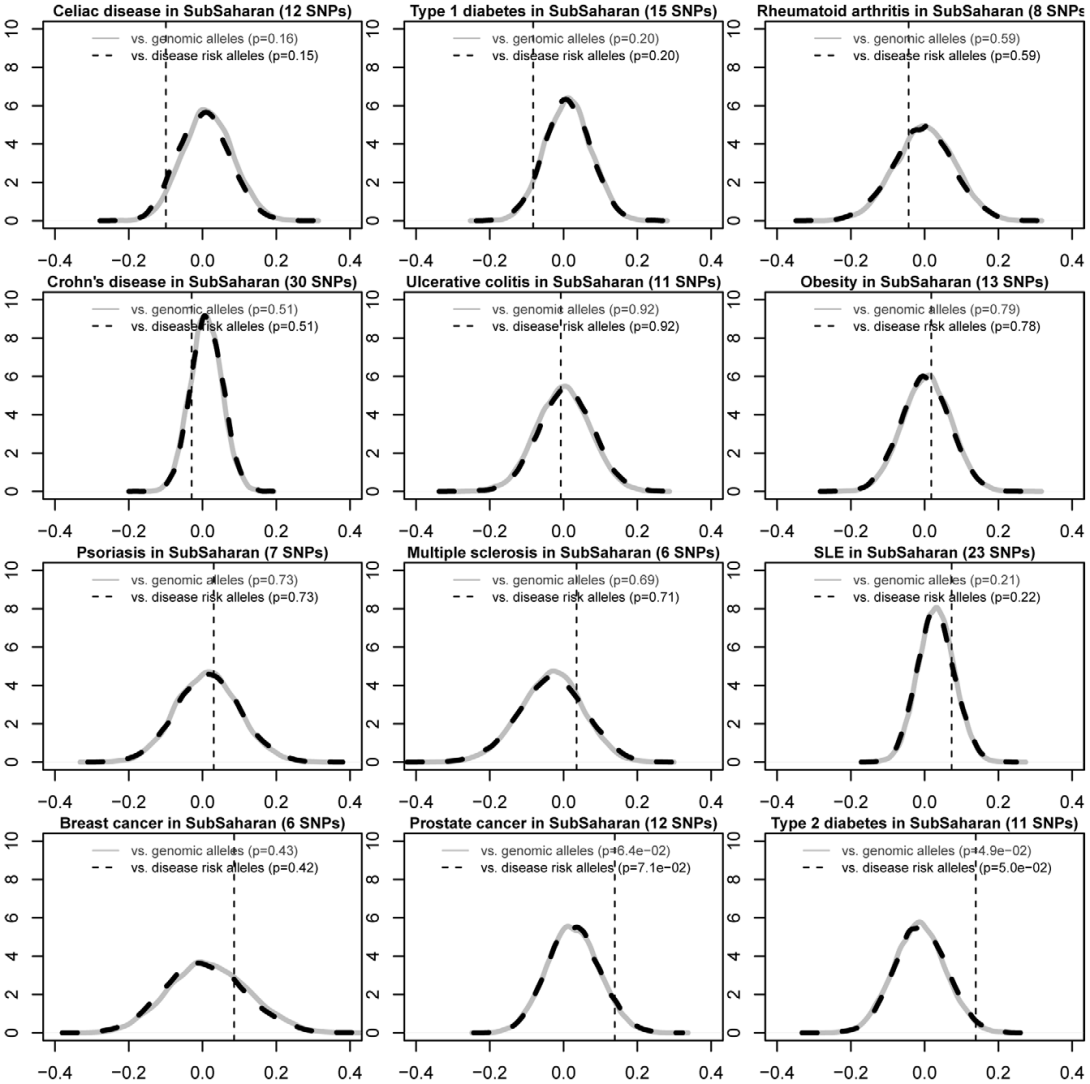
Differential risk allele frequencies in the Sub-Saharan African populations for 12 common diseases, compared with the frequencies in the European. Similar with Figure 4, we plotted the average increased RAFs in the Sub-Saharan Africa regions in the HGDP for each of 12 diseases, against the null distributions of frequency-matched control genomic alleles and risk alleles for other diseases. We ordered these 12 diseases by the increased RAF for a direct comparison. T2D shows significantly increased RAFs in the Sub-Saharan African populations, followed by prostate cancer. Two-side p values were calculated by comparing dotted vertical lines against the null distributions of frequency-matched control genomic alleles and risk alleles of other diseases. SNPs used in each figure were summarized in Table S4.

We used this method to systematically evaluate the directional differentiation of RAF of cross-ethnic risk alleles for all diseases in Varimed. We identified 12 common diseases that contain five or more independent cross-ethnic risk alleles (CEU R^2^<0.7); each of which had been known to associate with the disease with p<1×10^−6^ and be replicated in five or more different populations (Materials and Methods). T2D was the only disease that showed significantly decreased RAF in the East Asian populations (p = 1×10^−4^, Figure 4) and significantly increased RAF in the Sub-Saharan African populations (p = 4.9×10^−2^, Figure 5), compared with frequencies in the European populations. Prostate cancer showed relatively increased RAF in the Sub-Saharan African populations (p = 6.4×10^−2^, Figure 5), without decreased RAF in the East Asian populations ((p = 0.61, Figure 4). We repeated the study using disease-susceptible risk alleles that had been replicated in two independent papers with p<5×10^−8^, and got very similar results (Figures S4, S5, S6). One small difference is that prostate cancer showed significantly higher RAF in the Sub-Saharan African populations at replicated risk alleles (p = 4×10^−3^, Figure S6). Therefore, T2D risk alleles showed the most extreme differentiation of RAF across human populations, compared with other diseases.

Interestingly, ensembles of obesity-susceptible risk alleles did not show significant differentiation of RAF in either East Asian (p = 0.37, Figure 4) or Sub-Saharan African populations (p = 0.79, Figure 5). Eight out of 12 independent cross-ethnic obesity-susceptible risk alleles did not show significantly larger differentiation of RAF than control genomic alleles, individually (Figure S7). Among the remaining four risk alleles, two are located within *FTO* and known to increase the risk of T2D as well. Therefore, we did not observe any consistent pattern of RAF across obesity-susceptible risk alleles. The decoupling between obesity- and T2D-susceptible risk alleles indicates that their evolutionary histories are likely different.

### T2D showed the most significant population differentiation on Predicted Genetic Risk (PGR) among 40 diseases

Having shown the extreme differentiation of frequencies across T2D risk alleles, we further combined the effect size from all independent risk variants to systematically evaluate the differentiation of genetic risk of T2D and 39 other diseases. Using a method we had previously developed [22], [23], we calculated a Predicted Genetic Risk (PGR) of 40 diseases for each of 1,397 individuals using 1.46 million SNPs genotyped in the HapMap release 3 (HapMap3). We only used the HapMap3 data to calculate the PGR so that the same set of SNPs was used for each individual. For each disease, we identified risk SNPs that have been validated in five or more populations, estimated their increased Likelihood Ratio (LR) using the genotype frequencies in the case/control groups reported in each previous study, and finally combined the LRs from multiple SNPs using ethnic-specific linkage disequilibrium R^2^ data to report a summarized risk score for each disease. The summarized score estimates the Predicted Genetic Risk (PGR) for an individual given their genotypes at independent risk SNPs.

**Figure 6:**
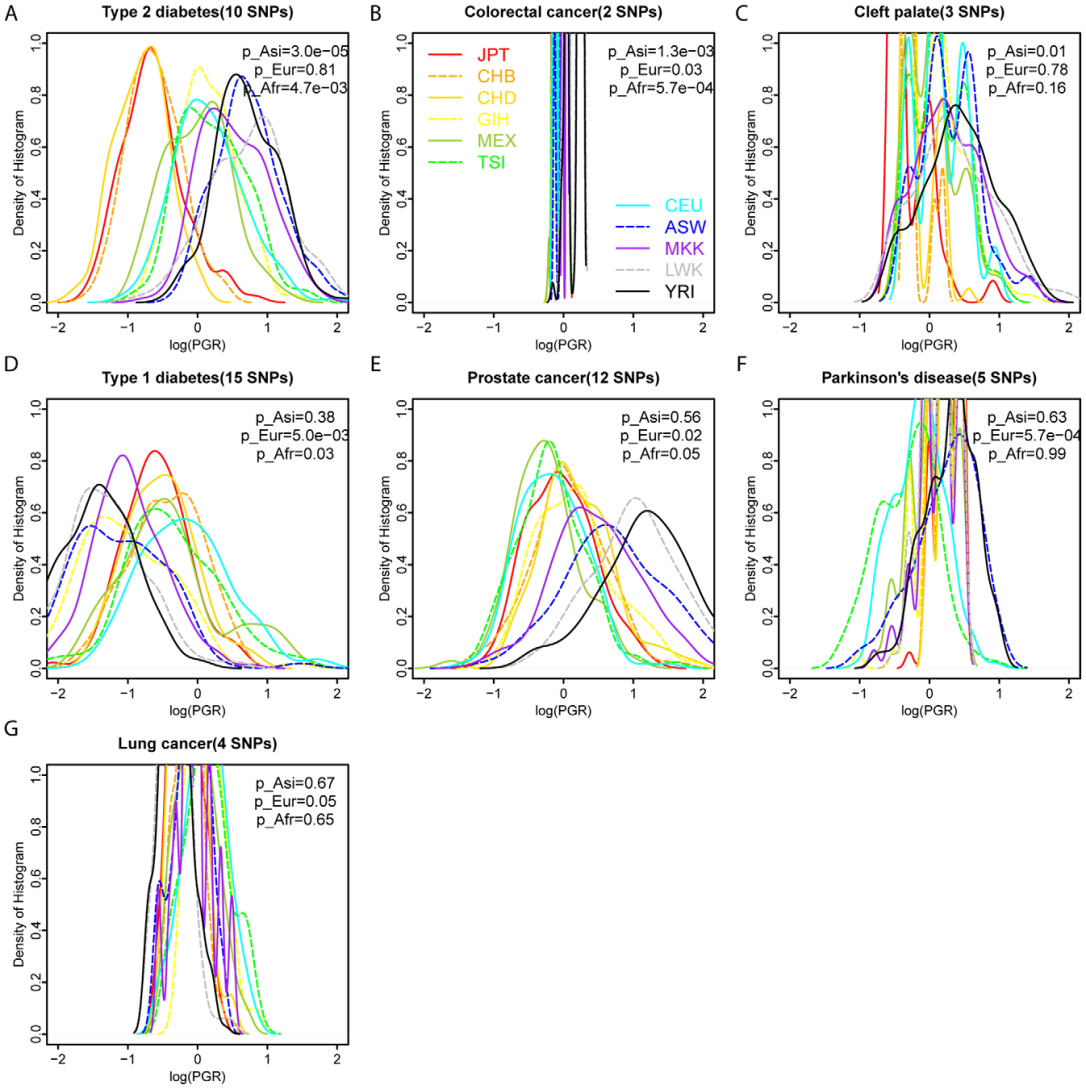
Seven diseases show significantly differential genetic risks across 11 HapMap populations, compared to frequency-matched control genomic genotypes. The density plots were drawn for the histograms of log(PGR) values with different colors and line styles representing each of 11 different HapMap3 populations. PGR represents Predicted Genetic Risk (PGR) with higher risk on the right. Seven diseases showed significantly differential PGR across populations, including type 2 diabetes (A), colorectal cancer (B), cleft palate (C), type 1 diabetes (D), prostate cancer (E), Parkinson's disease (F), and lung cancer (G). p_Afr shows the likelihood of observing lager log(PGR, African)-log(PGR, Other) values after randomly replacing disease genotypes with global frequency-matched genomic genotypes. For example, an average value of log(PGR) is 0.648 in African populations and −0.224 in other populations for T2D. After randomly replacing T2D genotypes with control genomic genotypes, there is only 4.7×10^−3^ chance finding an average value of log(PGR, African)-log(PGR, Other) larger than 0.872. Similarly, p_Asi and p_Eur represent the likelihoods of observing more extreme values of log(PGR, Asian)-log(PGR, Other) and log(PGR, European)-log(PGR, others) using randomly selected genomic genotypes, respectively. All p values were calculated as two-sided p values. SNPs used in each figure are summarized in Table S4.

We calculated the PGR for each of 1,397 individuals on 40 diseases (Table S5), and found that seven diseases showed significantly larger population differentiation than global frequency-matched genomic SNPs (Figure 6). After Benjamini-Hochberg multi-test correction, only T2D and colorectal cancer demonstrated significantly larger population differentiation than random genomic SNPs. After accounting for the genomic SNPs, T2D showed significantly increased PGR in the African populations relative to others (p = 4.7×10^−3^), and significantly decreased PGR in the Asian populations relative to others (p = 3.0×10^−5^). Colorectal cancer showed a similar pattern, but the PGR differences among population groups were much smaller because there were only two cross-ethnic SNPs. The statistical significance of observed PGR differences was estimated using a genomic background by randomly selecting global frequency-matched genomic SNPs and re-calculating the PGR on each disease using the random SNPs. We then calculated the likelihood of observing a larger PGR difference between each of three population groups (Asian, European, African) and others. Overall, T2D demonstrated the most significant population differentiation on PGR compared to frequency-matched genomic SNPs.

### Consistent pattern of T2D PGR using ethnic-specific SNPs, validated risk scores, and different genotyping/sequencing technologies

**Figure 7:**
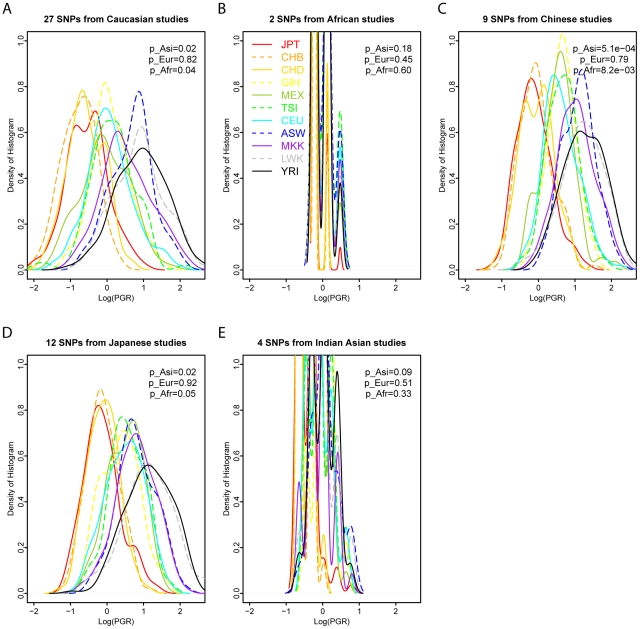
A consistent pattern of T2D PGR using SNPs and LRs from ethnic-specific studies. The density plots were drawn for the histograms of log(PGR) values across 11 HapMap3 population groups using T2D SNPs and likelihood ratios (LRs) from previous studies specifically on each of the following ethnicities: Caucasian (A), African (B), Chinese (C), Japanese (D), and Indian Asian (E). Eleven HapMap3 population groups are plotted with solid or dashed lines with different colors. PGR presents Predicted Genetic Risk with higher risk on the right. A consistent pattern was observed with the highest PGR in the African populations (LWK, YRI, ASW, MKK) and the lowest PGR in the Asian populations (JPT, CHD, CHB) regardless which ethnic-specific T2D SNPs and LRs were used. p_Asi, p_Eur, and p_Afr represent the likelihoods of observing more extreme values of log(PGR, Asian)-log(PGR, Other), log(PGR, European)-log(PGR, others), and log(PGR, African)-log(PGR, Other) using randomly selected control genomic genotypes, respectively. All p values were calculated as two-sided p values. SNPs used in each figure are summarized in Table S4.

Finally, we evaluated the robustness of the observed ethnic disparity of T2D PGR in three conditions. To evaluate the effect of SNPs discovered and validated in specific subpopulations, we compared the distributions of PGR using only the ethnicity-specific T2D SNPs, their genotype frequencies in the case and control groups, and LR separately from studies specifically on each of the following ethnicities: Caucasian, African, Chinese, Japanese, and Indian Asian (Figure 7). T2D PGR distributions demonstrated a strikingly similar pattern no matter which original studies were used to retrieve the previously discovered significant T2D SNPs, genotype frequencies, and LRs. African populations always have the highest PGR and Asian populations always have the lowest PGR.

**Figure 8:**
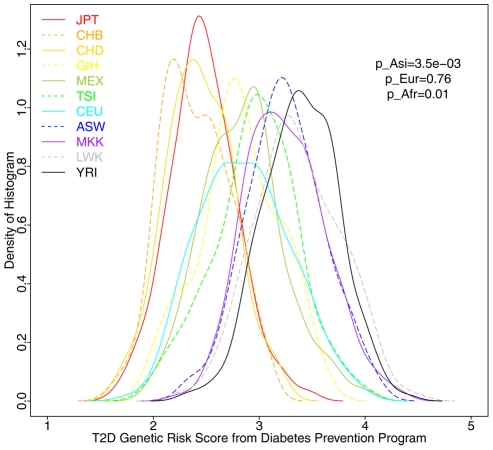
Distribution of T2D Genetic Risk Scores in 1,397 HapMap3 individuals. A high T2D Genetic Risk Score (GRS) had been previously shown to significantly associate with increased risk of developing diabetes in participants from Diabetes Prevention Program [19]. We calculated the GRS scores for 1,397 individuals using 20 SNPs measured in the HapMap and plotted the distribution of GRS across 11 HapMap populations. The distribution of GRS is very similar with that of PGR in Figure 6A. p_Asi, p_Eur, and p_Afr represent the likelihoods of observing more extreme values of log(GRS, Asian)-log(GRS, Other), log(GRS, European)-log(GRS, others), and log(GRS, African)-log(GRS, Other) using randomly selected control genomic genotypes, respectively. All p values were calculated as two-sided p values. GRS SNPs were summarized in Table S4.

Recently, a T2D genetic risk score had been validated to associate with the increased risk of developing diabetes in a prospective random trial on 2,843 Diabetes Prevention Program participants from five ethnic groups representative of the U.S. population [19]. The T2D genetic risk score was calculated by multiplying the number of risk alleles by the natural log of the odds ratio at each SNP, and summing over 34 SNPs. Twenty SNPs were measured in the HapMap3 individuals. We calculated the T2D risk score for each of 1,397 HapMap3 individuals, and found the same differential T2D genetic risks, with significantly increased risk in the African populations (p = 0.01) and significantly decreased risk in the Asian populations (p = 3.5×10^−3^, Figure 8).

**Figure 9:**
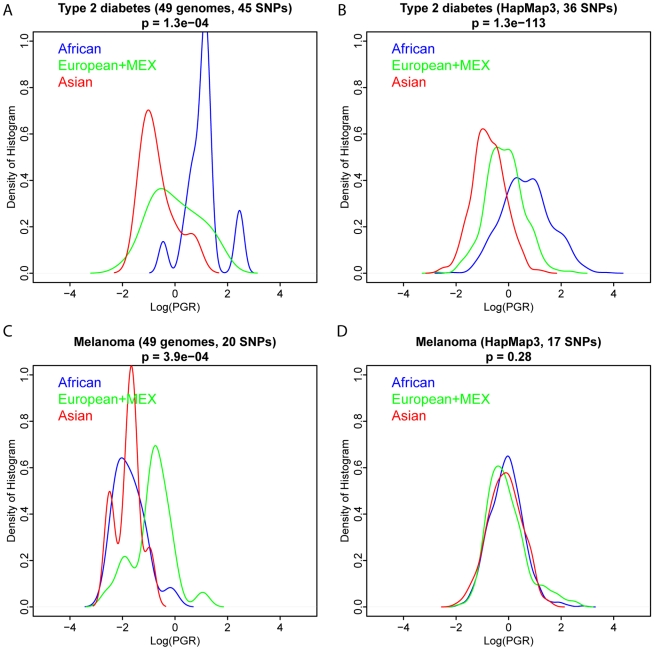
Comparison of PGR distributions between whole-genome sequencing and genotyping technologies. The ethnic distributions of log(PGR) of T2D and Melanoma were calculated for 49 individuals sequenced with over 55× coverage by Complete Genomics and 1,397 HapMap3 individuals genotyped on Illumina 1M and Affymetrix 6.0 arrays. Similar differential T2D genetic risks were observed between two technologies, while differential melanoma genetic risk was only observed from whole genome sequencing. SNPs used in each figure were summarized in Table S4.

Third, in each of the studies above, we were constrained in the number of SNPs usable for calculating PGR for T2D, based on intersecting the set of SNPs known to be associated with T2D with the set of SNPs genotyped in individuals in HapMap3 and HGDP. To ensure our findings are not an artifact of bias in the measured SNPs, we repeated our analysis using a larger set of T2D associated SNPs in 49 individuals for which whole genome sequencing is obtained, including 4 JPT, 4 CHB, 5 MEX, 3 Puerto Rican, 2 TSI, 14 CEU, 5 ASW, 1 MKK, 4 LWK, and 7 YRI. The subpopulation distributions of PGR were very similar between whole genome sequencing and genotyping technologies (HapMap3) for T2D (Figure 9). This equality did not hold for all diseases, as very different distributions are observed for melanoma. This suggests that current genotyping technology captures enough signals to reproduce the ethnic disparity for some common diseases, such as T2D, while whole genome sequencing will likely capture many more genetic variants influencing melanoma and perhaps other diseases.

## Discussion

We developed a novel method to systematically evaluate the directional differentiation of Risk Allele Frequencies (RAF) of ensembles of cross-ethnic SNPs for 12 common diseases across 11 populations from the HapMap project and 53 indigenous populations from the HGDP project. We found that type 2 diabetes (T2D) demonstrated the significant differentiation of RAF among diverse populations, compared with the European frequency-matched control genomic alleles and risk alleles for other diseases (Figure 3, Figure S4). T2D showed the most extreme differentiation among 12 common diseases, no matter whether we used cross-ethnic SNPs that had been replicated in five different populations (Figure 4, Figure 5) or SNPs that had been replicated in two studies (Figures S5, S6). This extreme differentiation is caused by the phenomenon that all T2D risk alleles share a consistent pattern of gradually decreasing population frequencies from Sub-Saharan Africa through Europe to East Asia regions (Figure 1, Figure 2, Figure S2).

This phenomenon, that T2D risk alleles decrease frequencies when humans migrate [24], suggests many potential explanations. One likely cause is the adaptation to the disparities of agriculture development across continents. It has been previously reported that some T2D SNPs have higher risk allele frequencies in populations where cereals are the main dietary component, and observed risk allele frequency might be related to historical events, such as the dispersal out of Sub-Saharan Africa to regions with different climates and the adoption of more specialized-often less diverse-diets (i.e. farming and animal husbandry vs. foraging) [25]. There were three major events in human evolution, including early migration from 200KYA to 10KYA, agriculture revolution and population expansion from 10KYA to 4KYA, and new world discovery and associated mass-migration and admixture after 4KYA [26]. The significantly decreased frequencies of T2D risk alleles in the East Asia might be caused by the agriculture revolution, including the cultivation of white rice and pork in China. A related explanation stems from the thrifty genotype [27] hypothesis, which asserts that a predisposition to insulin resistance may have protected individuals during periods of food deprivation by reducing muscle utilization of glucose and favoring glucose utilization in organs, such as brain, that operates through an insulin independent mechanism [27]. Combining these two related explanations together, we speculate that the decreasing T2D risk allele frequencies are caused by the promotion of energy storage and usage appropriate to environments and insistent energy intakes.

Another speculation is that T2D is known to find roots in the mismatch between our genetics and environment, as food contributes a significant environmental impact. When humans migrate, environmental change may have led to a mismatch between genetics and available diet, and put a positive evolutionary pressure on the frequencies of T2D protective alleles. Therefore, the decreasing T2D risk alleles are expected, while the other diseases are unusual given the underlying demographic history. Future evolutionary analysis on these T2D SNPs may provide some insight on the origin of this pandemic disease, as will more population-specific genetic studies.

Having shown the extreme differentiation of T2D RAF, we further combined the effect sizes from all independent risk variants and calculated a Predicted Genetic Risk (PGR) for each of 1,397 individuals in the HapMap3 project. T2D showed the most significant population differentiation among 40 diseases, after correcting for control genomic genotypes (Figure 6). We identified a consistent pattern of high PGR in the African and low PGR in the Asian regardless whether we used ethnic-specific SNPs (Figure 7), validated risk scores (Figure 8), or different genotyping/sequencing technologies (Figure 9). Our results indicate that there is indeed a differential T2D genetic risk across different populations across continents. The distributions we have found are very similar to a recent report measuring 19 common variants on five continent populations [15], with the highest risk in the African populations, and lowest risk in the East Asian populations.

The populations examined by this study are distributed broadly around the world, representing a wide range of environmental exposures and lifestyles. Hence, it is challenging to associate the increased prevalence of risk-associated alleles with actual manifestations of T2D, which we know to be heavily influenced by environmental factors. However, studies in England and the United States have consistently shown that individuals with African ancestry have increased diabetes rates relative to their neighbors of European or East Asian ancestry [28]–[31], while those with Chinese ancestry had lower incidence compared to others in a recent 10-year Canadian study [32]. At the same time, citizens in China have higher prevalence of T2D within their own country [33], [34]. Disparities in T2D rates may be attributed to social, cultural, and economic differences or possible genetic confounders such as admixing of ancestral ethnicities, though our results suggest that differential genetics may indeed play some role in these differences in incidence rates.

We also found that African had higher PGR on prostate cancer than other populations. Epidemiology data from Center for Disease Control and Prevention from 1999 to 2007 show that incidence of prostate cancer is 1.56 times higher in the African American than white American. Further investigation on the genetic reasons behind the observed ethnic disparity of disease incidence rates across ethnic/racial groups might identify personalized medicine to improve the health disparity.

Many challenges to evaluate the population differentiation of RAF and PGR remain. Foremost, many of the SNPs identified from genome-wide association studies (GWASs) are tag SNPs and are therefore not assumed to be causal [35]. However, each of the 12 cross-ethnic T2D SNP share the same risk allele and similar effect sizes across 34 different studied populations (Figure S1, Table S2), suggesting that they are the best representatives of the causal alleles based on the current data. The consistent observation of differential T2D genetic risk with different SNPs, risk scores, and technologies suggests validity as new causal variants are identified, but this remains a hypothesis that needs to be tested in the future. Second, we acknowledge that we adopted a relaxed p value cutoff of p<1×10^−6^ to identify cross-ethnic SNPs for a wide-variety of diseases for comparisons. With more GWAS in diverse population groups, a more rigorous cutoff and ethnicity-specific effects should be used. Third, there may be some ethnic-specific gene-environment interaction. Forth, our observed disparity of PGR between population groups might be related to the disparities in the application of modern genetic tools to study diseases across ethnicities. Finally, a large component of heritable risk is still missing for most common diseases, and consequently missing in our analysis here [36]. Future GWAS and sequencing studies on different ethnic groups under diversified environmental conditions will likely further reveal and illustrate the origins of complex diseases.

In conclusion, we found that T2D risk alleles demonstrated extreme differentiation compared to other diseases, with population frequencies decreasing from Sub-Saharan Africa and through Europe to East Asia. These patterns may contribute to the observed disparity of T2D incidence rates across worldwide ethnic populations.

## Materials and Methods

### VARiant Informing MEDicine (Varimed): A quantitative disease–SNP association database

As described previously [6], we have been manually curating a quantitative human disease-SNP association database from literature. First, we downloaded all abstracts from MEDLINE, and identified human genetic papers using a list of Medical Subject Headings [37], such as “Genome-Wide Association Study”, “Genetic Variation”, “Polymorphism, Genetic”, “Genome, Human”, “Polymorphism, Single Nucleotide”, “Genotype”, “Genetic Predisposition to Disease”, “Case-Control Studies”, “Alleles”, “Cohort Studies”. We then filtered these papers through abstracts, titles, and keywords. We also downloaded papers from curated disease-SNP databases, such as GWAS catalog from National Human Genome Research Institute [38]. Combined together, we identified 5,065 human genetic papers representing 1,495 diseases. Second, four curators manually extracted data from the full text, figures, tables, and supplemental materials of 5,065 human genetics papers, and recorded more than 100 features from each paper. We recorded many aspects of the associations, including the disease name (e.g. coronary artery disease), specific phenotype (e.g. acute coronary syndrome in coronary artery disease), study population (e.g. Finnish individuals), case and control population (e.g. 2,508 patients with coronary artery disease proven by angiography), gender distribution, genotyping technology, major/minor/risk alleles, odds ratio, 95% confidence interval of the odds ratio, published p value, and genetic model. Studies on similar diseases were categorized and mapped to the Concept Unique Identifiers (CUI) from the Unified Medical Language System (UMLS) [39]. For each study, the frequencies of each genotype and allele in the case and control populations were recorded, and used to estimate the effect size [22], [23].

### Systematically identify independent cross-ethnic and replicated risk alleles for 12 diseases

We identified all disease-susceptible alleles from Varimed that were reported to increase the risk of human disease with p-value<10^−6^. We focused on alleles that were directly associated with the increased risk of human disease by removing the following SNPs: SNPs that were specified as non-significant in the original papers, SNPs that were identified from studies with diseased patients in the control groups, SNPs that were associated with non-disease traits, SNPs that were associated with disease through haplotype blocks or interaction terms. Risk alleles on the negative strands were translated into alleles in the positive strands. Negative strands were identified by comparing the major/minor alleles in the study with the major/minor alleles in the similar population in the HapMap3. Many risk alleles had been reported in multiple studies, and we integrated all studies and ranked the risk alleles by the strength of replication, including the number of replicated populations, studies, and total sample sizes.

We identified cross-ethnic SNPs as SNPs being associated with a disease with a p-value<1×10^−6^ in at least one populations and reported as significant in five or more different subpopulations with a p-value<1×10^−6^ in GWAS studies or p-value<0.01 in small candidate studies. We removed SNPs that were specified as non-significant in the original papers. For each disease, we ranked all cross-ethnic SNPs by the number of replication studies, the total sample sizes and the number of populations where the associations being replicated. Starting from risk alleles with the strongest evidence, we identified risk alleles with the linkage disequilibrium R^2^≥0.7 in the CEU population in the HapMap project, and removed ones with less evidence. We identified 12 diseases with 5 or more independent cross-ethnic risk alleles, each of them being validated in five or more populations. We found 12 independent cross-ethnic risk alleles for Type 2 diabetes (T2D), and plotted their association p values across 34 populations using the levelplot function in R. We then evaluated the between-study heterogeneity of allelic odds ratios on each of 12 cross-ethnic T2D SNPs using the meta R package. Similarly, we identified disease SNPs that have been replicated in two or more papers with p<5×10^−8^.

### Evaluate the population differentiation of RAF against European frequency-matched control genomic alleles at individual disease-susceptible SNP

We retrieved the allele frequencies at 2.3 million SNPs in the 11 populations from HapMap 2+3, which were released in August 2010. We plotted the RAFs across the 11 populations as bar graphs at each independent cross-ethnic risk allele for each disease using a barplot function in R.

We then evaluated the statistical significance of the population differentiation of RAF at each disease SNP by calculating the percentage of European frequency-matched control genomic alleles that show the RAF difference larger than the observed. For each risk allele, we retrieved all control genomic alleles sharing similar average frequencies within ±0.01 in the European (TSI, CEU) populations. Then, we calculated the percentage of matched genomic alleles that have both African frequencies higher than the observed and Asian frequencies lower than the observed. We recorded the percentage as a p value which is the likelihood of finding similar or larger differentiation of RAF from frequency-matched genomic alleles. All risk alleles with p<0.05 were considered as showing significantly larger population differentiation than control genomic alleles.

We received the measured and imputed allele frequencies at 3.1 million SNPs across 53 populations from 1,064 individuals from Human Genome Diversity Panel (HGDP) from Joseph Pickrell from University of Chicago [7], [13]. We modified a script from Joseph Pickrell using Generic Mapping Tools [40] and plotted the worldwide map showing the distribution of RAF across the 53 HGDP populations [13]. Similar with the analysis on HapMap, we calculated the percentages of European frequency-matched genome alleles that have both Sub-Saharan African frequencies higher than the observed and East Asian frequencies lower than the observed.

We categorized populations into three major regions. Sub-Saharan Africa region includes BantuSouthAfrica, Biaka Pygmies, Mandenka, Mbuti Pygmies, San, Yoruba, and BantuKenya. East Asia region includes Cambodian, Dai, Daur, Han, Han-NChina, Hezhen, Japanese, Lahu, Miao, Mongola, Naxi, Oroqen, She, Tu, Tujia, Uygur, Xibo, Yakut, and Yi. Europe region includes Adygei, Basque, French, Italian, Orcadian, Russian, Sardinian, and Tuscan.

### Compare F*_ST_* values of T2D SNPs against those of control genomic SNPs

Global F*_ST_*, as well as three pairwise F*_ST_* (African vs. East Asian, African vs. European, East Asian vs. European) values were calculated for all SNPs from HapMap3, using a custom script implementing the method in the PopGen module of BioPerl [41]. For the calculations, all populations for the respective geographic regions were pooled, so that the global F*_ST_* reflected the overall differentiation of those major geographic regions. All SNPs were grouped into 10 bins according to their average Minor Allele Frequencies (MAF) across the European HapMap3 populations (CEU, TSI), and T2D SNPs were compared to all genomic SNPs within the same MAF bin. For the Mann-Whitney U test, we used normalized F*_ST_* values, which were obtained by subtracting the mean and dividing by the standard deviation within each MAF bin for each SNP.

### Systematically evaluate directional population differentiation of ensembles of independent cross-ethnic risk alleles for 12 diseases

We evaluated the population differentiation of an ensemble of 10 independent cross-ethnic T2D risk alleles by comparing their average increased RAF in the African populations against the null distribution of control genomic alleles and 15,649 risk alleles from 975 diseases. First, we randomly retrieved 10 control genomic alleles that share similar European frequencies (±0.05) with T2D risk alleles, and calculated their average increased frequencies in the African populations (MKK, LWK, YRI, ASW). Repeating the above process 10,000 times, we drew a null distribution of increased frequencies between African and European populations from control genomic alleles. The average increased frequency of 10 T2D risk alleles in the African vs. European populations was then compared with the null distribution to calculate the likelihood of observing consistently increased African frequencies from matched genomic alleles.

Similarly, we randomly retrieved 10 European frequency-matched alleles (±0.05) from 15,649 risk alleles from other diseases, and calculated a null distribution of increased African frequencies from disease risk alleles. We also calculated the null distribution of Asian frequencies from HapMap, East Asian and Sub-Saharan African frequencies from HGDP using European frequency-matched genomic alleles and risk alleles from other diseases.

### Calculate the distribution of PGR across the 11 HapMap3 population groups

Using a method we described previously [22], [23], we calculated the Predicted Genetic Risk (PGR) of 40 diseases for 1,397 individuals from HapMap release 3. Each individual was genotyped at 1.46 million SNPs. We calculated the PGR of 40 diseases using independent cross-ethnic SNPs, each of them had been validated in five or more different populations. We estimated a genetic risk using a likelihood ratio for each SNP defined by the relative frequency of an individual's genotype in the diseased vs. healthy control populations (e.g., given a genotype “AA”, LR = Pr(AA|diseased)/Pr(AA|control)). The LR incorporated both the sensitivity and specificity of the test and provided a direct estimate of how much a test result would change the odds of having a disease [23]. We excluded studies with diseased patients in the control group. For each allele, we averaged the LRs from multiple studies with a weight of the square root of the sample size to give higher confidence to studies with larger sample size. For SNP pairs in linkage disequilibrium (R^2^≥0.3 in the corresponding population group), we removed the SNP with weaker evidence according to the number of replication studies, sample sizes and validated populations. We considered remaining SNPs as independent genetic test and multiplied their LRs to report the summarized score as the PGR. We then plotted the distribution of PGR across 11 HapMap populations using a kernel density function in the R package.

### Evaluate the statistical significance of population differentiation of PGR against control genomic SNPs

To evaluate the statistical significance of the population differentiation of PGR after correcting the difference of RAF, we randomly replaced disease genotypes with global frequency-matched non-associated genotypes, and re-calculated the PGR for each of 1,397 HapMap individuals. We calculated the PGR using the random control genomic genotypes along with the original LR, averaged the log(PGR) values for each of 11 population groups, and repeated the process 100,000 times. Then we calculated the p_Afr as the percentage of obtaining a value of log(PGR, African)-log(PGR, Other) from random genotypes larger than the observed values. Similarly, we calculated p_Asi and P_Eur as the likelihood of obtaining more extreme values of log(PGR, Asian)-log(PGR, Other) and log(PGR, European)-log(PGR, Other) from random genomic genotypes. Each p value was calculated as a two-side p value.

### Calculate the distribution of PGR using SNPs and LRs from ethnic-specific studies

We recalculated the distribution of PGR across the 11 HapMap populations using SNPs and LRs that were associated with T2D with p<1×10^−6^ in the original studies on each of the following populations, including Caucasian, African, Chinese, Japanese, and Indian Asian.

### Whole-genome sequences of 49 individuals from 10 population groups

Whole-genome sequences were produced using published methods [42] for 49 cell-line derived DNA samples obtained from the Coriell Institute. Each genome was sequenced to over 55× coverage and calls were produced using a local *de novo* assembly based pipeline. Comparison of these data to dbSNP was performed using alignment-based methods which account for SNPs which may be contained within more complex variant sequences.

We pooled the 49 samples into three groups: Asian, African, and Others. Then we calculated the distribution of PGR across these three population groups on T2D and Melanoma. All disease SNPs with p<1×10^−6^ in the original studies on any ethnicity were used.

## Supporting Information

Figure S1 Each of 12 cross-ethnic T2D SNPs shares the same risk allele and similar odds ratio in 34 studied populations. The heatmap graph summarizes the odds ratios across 34 studied populations at 12 independent cross-ethnic T2D SNPs. Each row represents a T2D SNP replicated in five or more populations. For example, 7903146_T_TCF7L2 represents a T risk allele at rs7903146 in *TCF7L2*. All odds ratios are larger than 1 with the risk allele listed at the left. For any SNP pairs with linkage disequilibrium R^2^≥0.7 in HapMap Caucasian, only the SNP with stronger evidence is kept. Two SNPs are considered as independent in *TCF7L2*, *CDKAL1*, and *KCNQ1* because their R^2^s are 0.512, 0.677, and 0.425 respectively. Detailed evidence including PubMed and p values in each population is shown in Table S2. Meta-analysis indicates that the study group consists of at least two populations from distinct population groups, such as East Asian, European, African, Mexican, and Indian Asian.
(PDF)

Figure S2 HGDP data show that T2D risk alleles decrease frequencies from Sub-Saharan Africa to East Asia (five SNPs not listed in Figure 2). Risk allele frequencies (RAF) are shown as dark blue wedges across the 53 HGDP populations at five T2D SNPs that are not listed in Figure 2. The frequencies of protective allele are shown as orange wedges. For each T2D risk allele, a p value was calculated as the percentage of genomic alleles with matched frequencies in European populations that showed both higher frequencies in the Sub-Saharan Africa regions and lower frequencies in the East Asia regions than the observed RAF. The (anc) in the sub-title indicates that the risk allele is the ancestral allele according to mammalian sequence data, retrieved from the dbSNP.
(PDF)

Figure S3 *F*_ST_ values of T2D SNPs against European frequency-matched control genomic SNPs in HapMap3. For each multiethnic T2D SNP, global and three pairwise (African vs. East Asian, African vs. Europe, East Asian vs. Europe) *F*_ST_ values were calculated and compared with the *F*_ST_ distribution of genomic SNPs that were within the same 5% European minor allele frequency (MAF) bins. Rank percentiles are shown for T2D SNPs against MAF matched genomic SNPs. All T2D SNPs show elevated *F*_ST_ values, with five out of ten T2D SNPs among 10% and 1 (rs11196205 in *TCF7L2*) among the top 1% of the empirical distribution for at least one of four population comparisons. P values were calculated to compare the *F*_ST_ values of T2D SNPs against frequency matched genomic SNPs, and listed at the top (Mann-Whitney U test).
(PDF)

Figure S4 Directional population differentiation of RAF for T2D SNPs replicated in two papers. Eleven independent SNPs had been replicated to associate with T2D with p<5×10^−8^ in two distinct papers. Ten of them had been measured in the HapMap project, except rs5219. They were combined as an ensemble to calculate the average increased RAFs in the Asia (A) and Africa (B) populations from HapMap, and the East Asia (C) and Sub-Saharan Africa (D) populations from HGDP, compared with the RAFs in the European populations. The average increased RAFs of T2D risk alleles are shown as dotted vertical lines, compared against the null distributions of average increased RAFs of 10 alleles randomly drawn from genomic alleles (solid black curve) and disease-susceptible risk alleles (dashed grey curve) that share the same allele frequencies with T2D risk alleles in the European populations. Two-side p values were calculated by comparing dotted vertical lines against the null distributions of frequency-matched genomic alleles and disease-susceptible risk alleles. SNPs used in each figure are summarized in Table S4.
(PDF)

Figure S5 Differential RAF in the East Asian versus European populations at replicated SNPs for 12 common diseases. For each of 12 diseases, we identified SNPs that had been replicated for association with p<5×10^−8^ in two distinct papers. We calculated their average increased RAF (dotted vertical line) in the East Asian versus European populations in HGDP, compared with the null distributions of European-frequency-matched control genomic alleles (solid density plot) and risk alleles for other diseases (dotted density plot). These 12 diseases were ordered by the increased RAF in the East Asian populations. T2D is the only disease showing significantly lower RAF in the East Asian populations. SNPs used in each figure were summarized in Table S4.
(PDF)

Figure S6 Differential RAF in the Sub-Saharan African versus European populations at replicated SNPs for 12 common diseases. For each of 12 diseases, we identified SNPs that had been replicated for association with p<5×10^−8^ in two distinct papers. We calculated their average increased RAF (dotted vertical line) in the Sub-Saharan African versus European populations in HGDP, compared with the null distributions of European-frequency-matched genomic control alleles (solid density plot) and risk alleles for other diseases (dotted density plot). These 12 diseases were ordered by the increased RAF in the Sub-Saharan African populations. Prostate cancer and T2D shows significantly increased RAFs in the Sub-Saharan African populations. SNPs used in each figure were summarized in Table S4.
(PDF)

Figure S7 RAF of Obesity risk alleles across the 11 HapMap populations. The frequencies of 11 independent multiethnic Obesity risk alleles are shown as heights of bars across the 11 HapMap populations. RAFs in the Asian populations (JPT, CHB, CHD) are shaded and listed on the left. RAFs in the African populations (ASW, MKK, LWK, YRI) are colored in grey and listed on the right. Higher RAFs in the African and lower RAFs in the Asian populations are only observed in 2 SNPs in *FTO* and 2 SNPs outside the gene region, including rs29941 and rs925946. Two SNPs in *FTO* are shared genetic risk variants with T2D. No consistent patterns are observed across obesity-specific risk alleles. The (anc) in the sub-title indicates that the risk allele is the ancestral allele from mammalian data, retrieved from the dbSNP.
(PDF)

Table S1 Significance of T2D association and replication of the 12 cross-ethnic SNPs.
(PDF)

Table S2 Associations of 12 independent cross-ethnic T2D SNPs across 34 populations.
(PDF)

Table S3 Between-study heterogeneity analysis of allelic Odds Ratios on T2D risk alleles.
(PDF)

Table S4 Summary of disease-susceptible SNPs used in the figures.
(PDF)

Table S5 Statistical significance of ethnic disparity of PGR across 11 HapMap3 populations on 40 diseases.
(PDF)
