# Supplementary figures and images for: Type 2 Diabetes Risk Alleles Demonstrate Extreme Directional Differentiation among Human Populations, Compared to Other Diseases
Source: PLoS Genet. 2012 Apr 12;8(4):e1002621. doi: 10.1371/journal.pgen.1002621 (PMC3325177; doi:10.1371/journal.pgen.1002621)

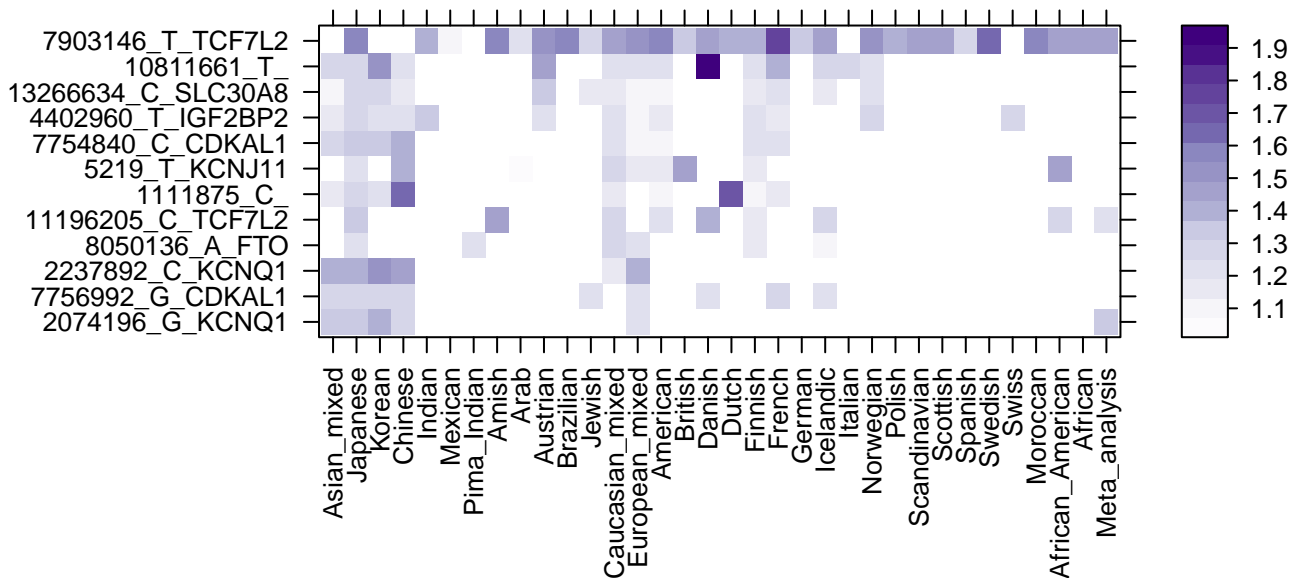

Supplement: Figure S1 — Each of 12 cross-ethnic T2D SNPs shares the same risk allele and similar odds ratio in 34 studied populations. The heatmap graph summarizes the odds ratios across 34 studied populations at 12 independent cross-ethnic T2D SNPs. Each row represents a T2D SNP replicated in five or more populations. For example, 7903146_T_TCF7L2 represents a T risk allele at rs7903146 in TCF7L2. All odds ratios are larger than 1 with the risk allele listed at the left. For any SNP pairs with linkage disequilibrium R2≥0.7 in HapMap Caucasian, only the SNP with stronger evidence is kept. Two SNPs are considered as independent in TCF7L2, CDKAL1, and KCNQ1 because their R2s are 0.512, 0.677, and 0.425 respectively. Detailed evidence including PubMed and p values in each population is shown in Table S2. Meta-analysis indicates that the study group consists of at least two populations from distinct population groups, such as East Asian, European, African, Mexican, and Indian Asian. (PDF) [file pgen.1002621.s001.pdf]

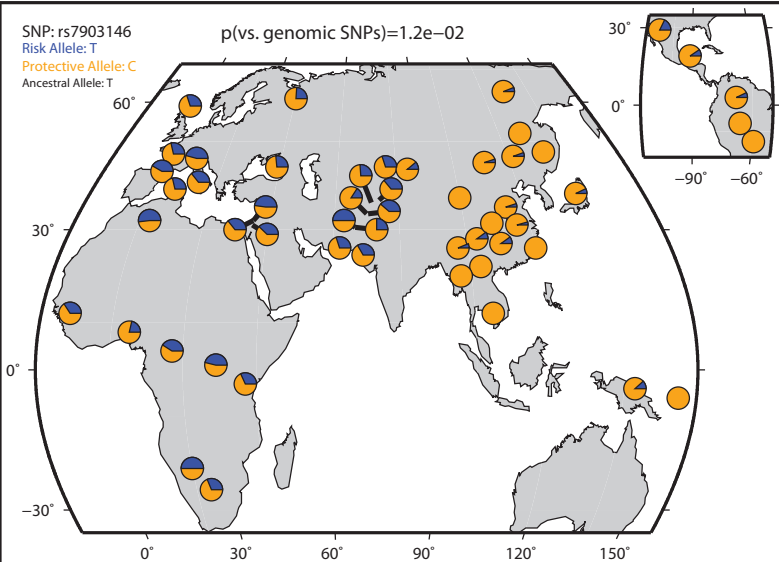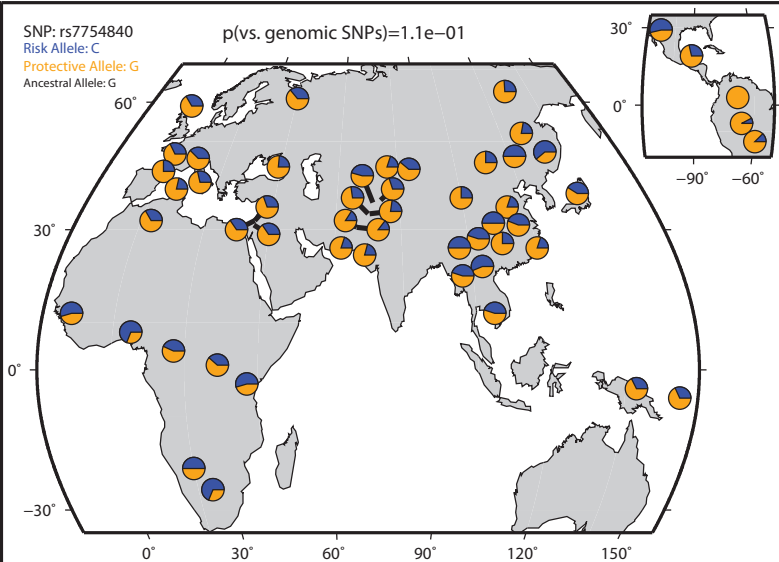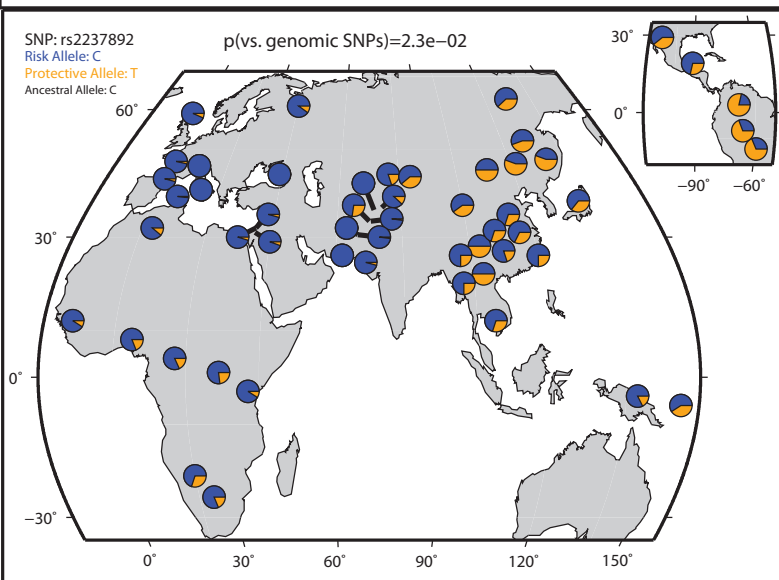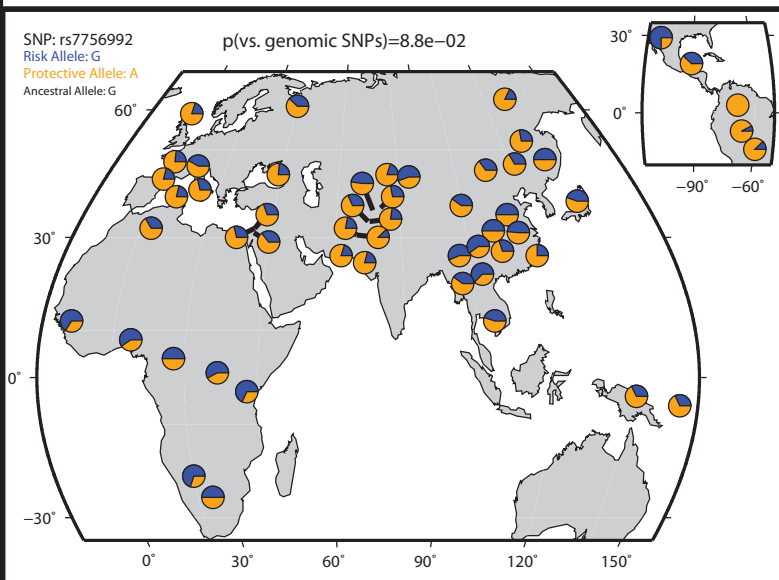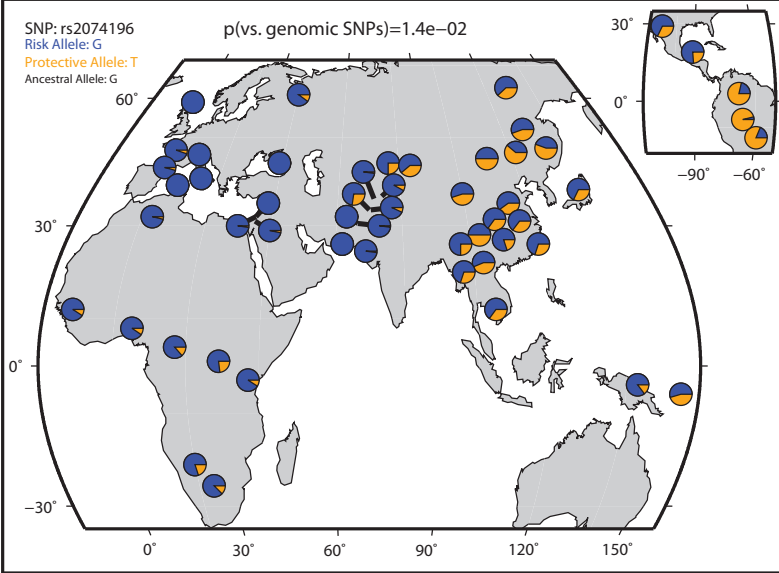

Supplement: Figure S2 — HGDP data show that T2D risk alleles decrease frequencies from Sub-Saharan Africa to East Asia (five SNPs not listed in Figure 2). Risk allele frequencies (RAF) are shown as dark blue wedges across the 53 HGDP populations at five T2D SNPs that are not listed in Figure 2. The frequencies of protective allele are shown as orange wedges. For each T2D risk allele, a p value was calculated as the percentage of genomic alleles with matched frequencies in European populations that showed both higher frequencies in the Sub-Saharan Africa regions and lower frequencies in the East Asia regions than the observed RAF. The (anc) in the sub-title indicates that the risk allele is the ancestral allele according to mammalian sequence data, retrieved from the dbSNP. (PDF) [file pgen.1002621.s002.pdf]

global (p = 0.0057)

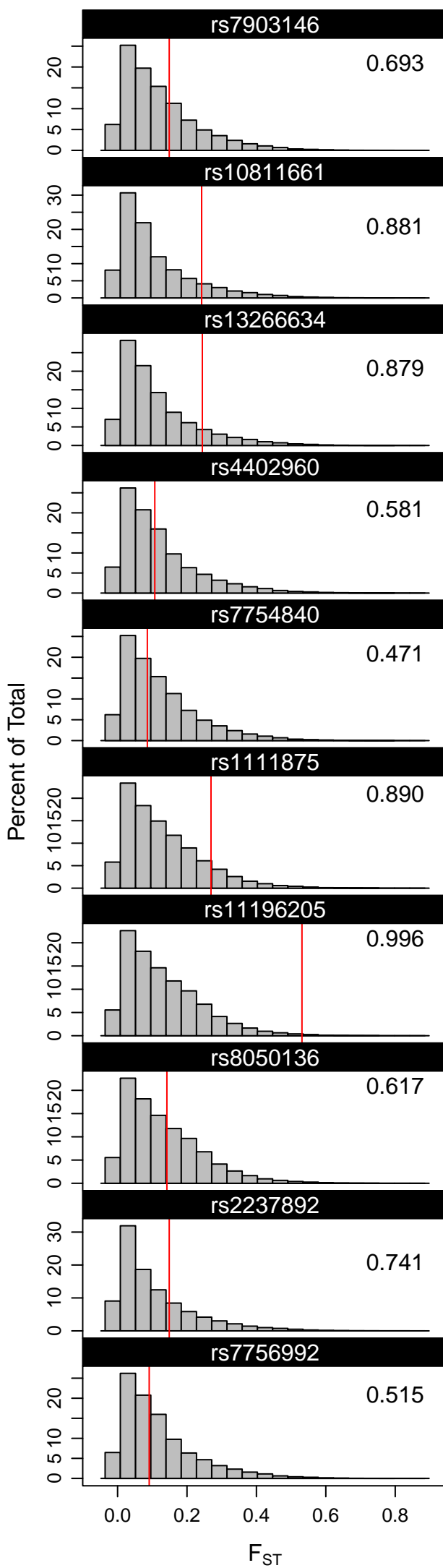

africa\_eastAsia (p = 0.0041)

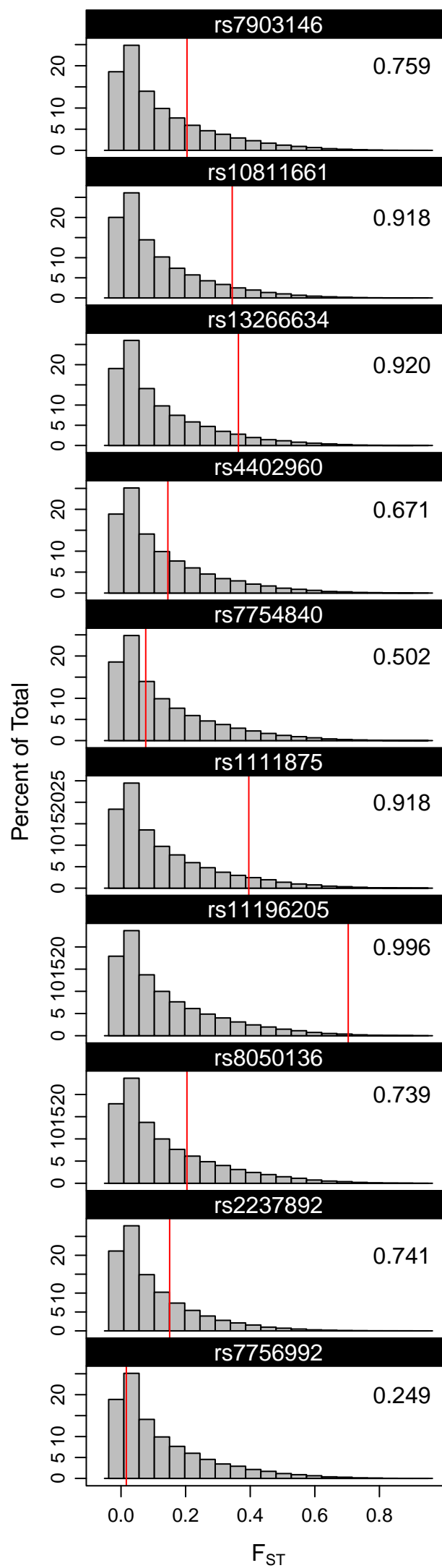

africa\_europe (p = 0.3)

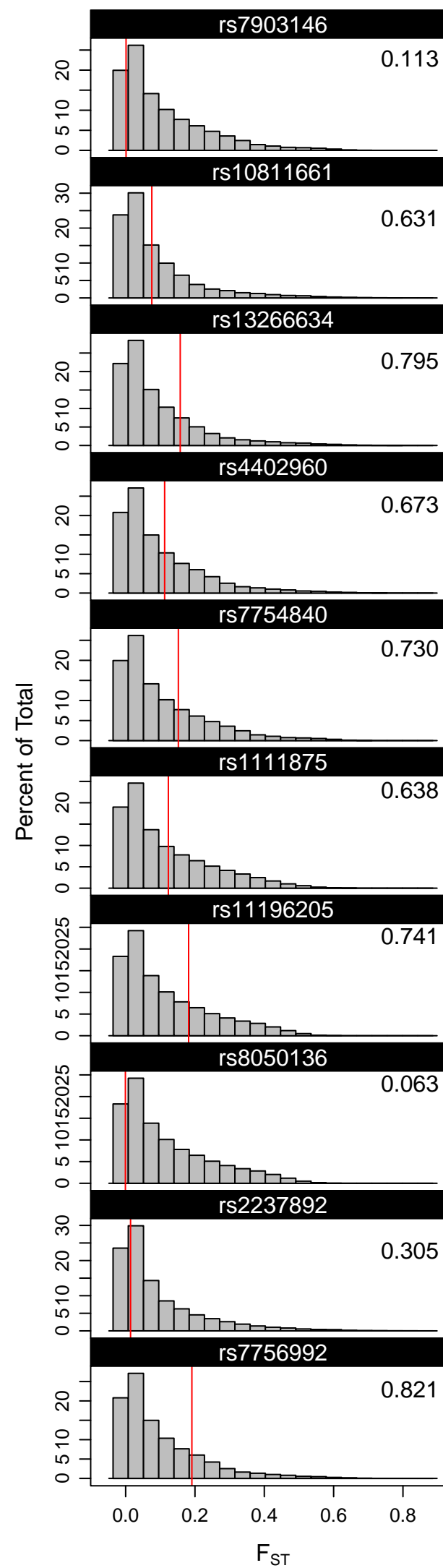

eastAsia\_europe (p = 0.025)

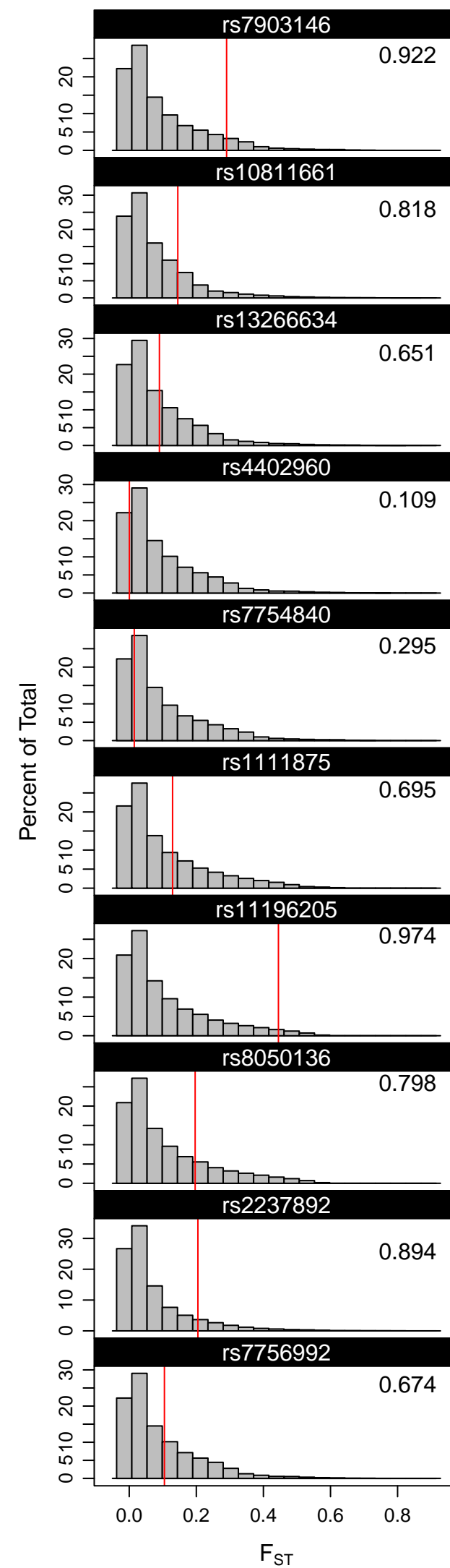

Supplement: Figure S3 — F ST values of T2D SNPs against European frequency-matched control genomic SNPs in HapMap3. For each multiethnic T2D SNP, global and three pairwise (African vs. East Asian, African vs. Europe, East Asian vs. Europe) F ST values were calculated and compared with the F ST distribution of genomic SNPs that were within the same 5% European minor allele frequency (MAF) bins. Rank percentiles are shown for T2D SNPs against MAF matched genomic SNPs. All T2D SNPs show elevated F ST values, with five out of ten T2D SNPs among 10% and 1 (rs11196205 in TCF7L2) among the top 1% of the empirical distribution for at least one of four population comparisons. P values were calculated to compare the F ST values of T2D SNPs against frequency matched genomic SNPs, and listed at the top (Mann-Whitney U test). (PDF) [file pgen.1002621.s003.pdf]

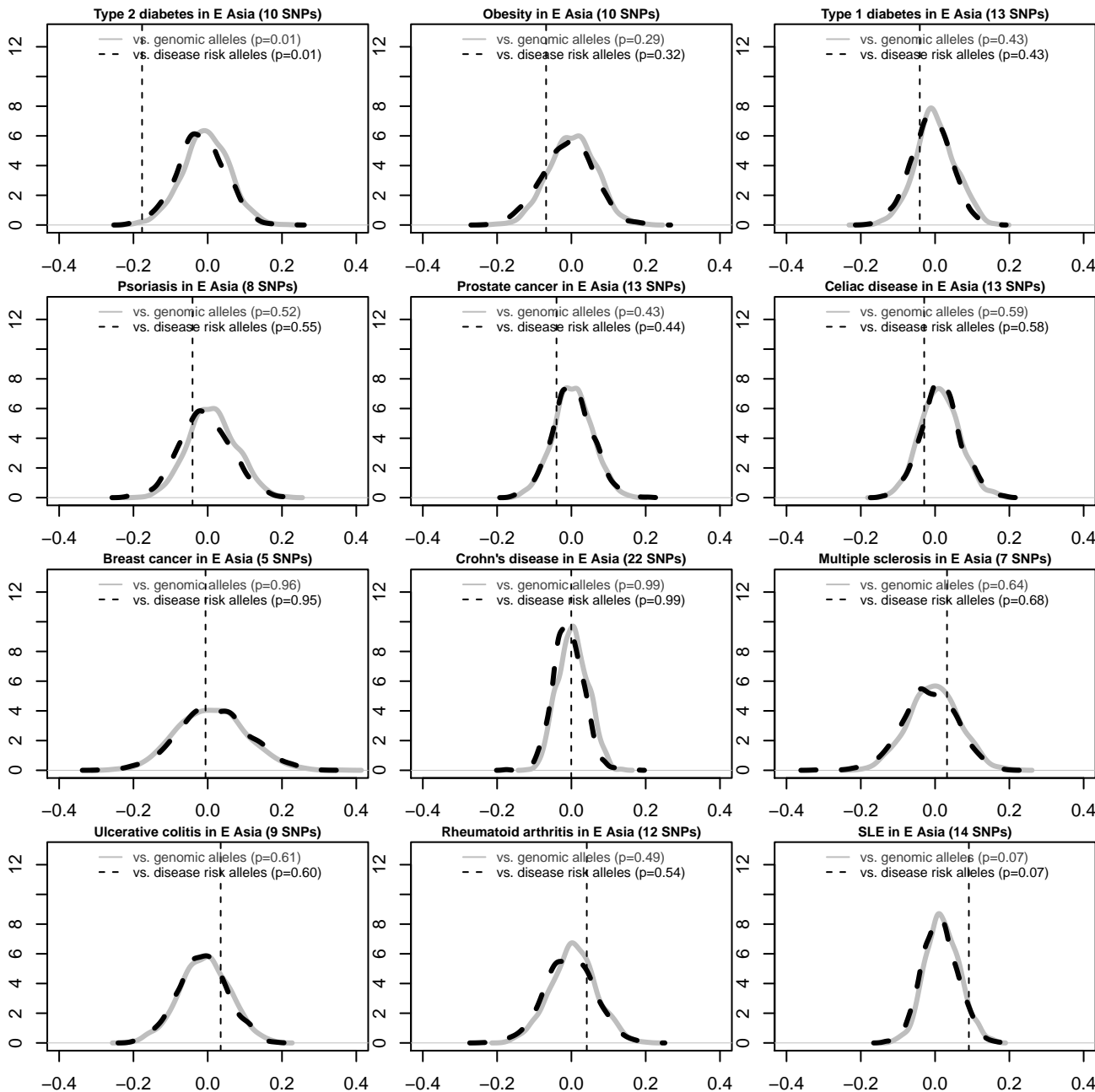

Supplement: Figure S5 — Differential RAF in the East Asian versus European populations at replicated SNPs for 12 common diseases. For each of 12 diseases, we identified SNPs that had been replicated for association with p<5×10^−8^ in two distinct papers. We calculated their average increased RAF (dotted vertical line) in the East Asian versus European populations in HGDP, compared with the null distributions of European-frequency-matched control genomic alleles (solid density plot) and risk alleles for other diseases (dotted density plot). These 12 diseases were ordered by the increased RAF in the East Asian populations. T2D is the only disease showing significantly lower RAF in the East Asian populations. SNPs used in each figure were summarized in Table S4. (PDF) [file pgen.1002621.s005.pdf]

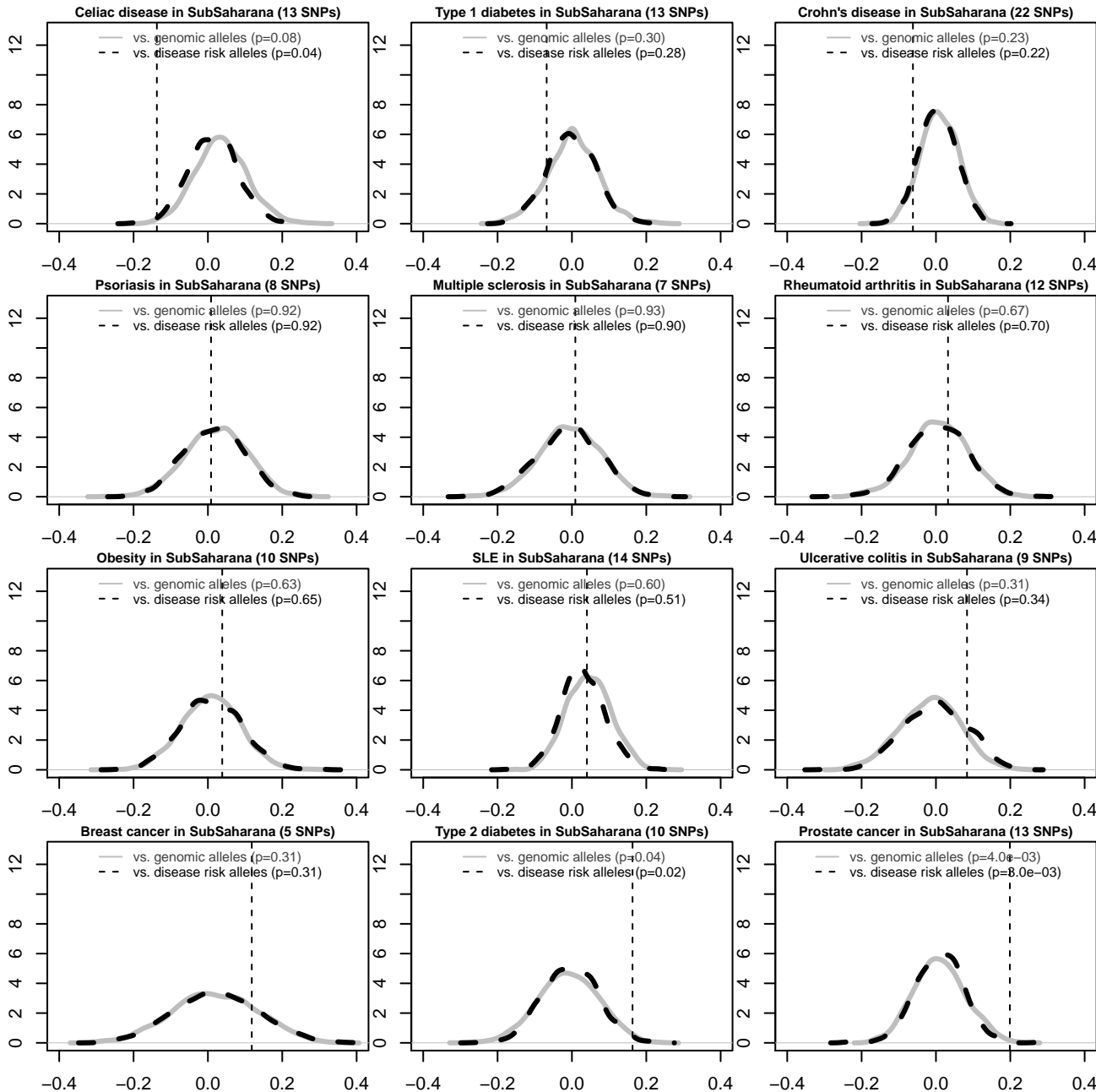

Supplement: Figure S6 — Differential RAF in the Sub-Saharan African versus European populations at replicated SNPs for 12 common diseases. For each of 12 diseases, we identified SNPs that had been replicated for association with p<5×10^−8^ in two distinct papers. We calculated their average increased RAF (dotted vertical line) in the Sub-Saharan African versus European populations in HGDP, compared with the null distributions of European-frequency-matched genomic control alleles (solid density plot) and risk alleles for other diseases (dotted density plot). These 12 diseases were ordered by the increased RAF in the Sub-Saharan African populations. Prostate cancer and T2D shows significantly increased RAFs in the Sub-Saharan African populations. SNPs used in each figure were summarized in Table S4. (PDF) [file pgen.1002621.s006.pdf]
