# Supplementary material for: Type 2 Diabetes Risk Alleles Demonstrate Extreme Directional Differentiation among Human Populations, Compared to Other Diseases
Source: PLoS Genet. 2012 Apr 12;8(4):e1002621. doi: 10.1371/journal.pgen.1002621 (PMC3325177; doi:10.1371/journal.pgen.1002621)
Supplement: Figure S4 — Directional population differentiation of RAF for T2D SNPs replicated in two papers. Eleven independent SNPs had been replicated to associate with T2D with p<5×10^−8^ in two distinct papers. Ten of them had been measured in the HapMap project, except rs5219. They were combined as an ensemble to calculate the average increased RAFs in the Asia (A) and Africa (B) populations from HapMap, and the East Asia (C) and Sub-Saharan Africa (D) populations from HGDP, compared with the RAFs in the European populations. The average increased RAFs of T2D risk alleles are shown as dotted vertical lines, compared against the null distributions of average increased RAFs of 10 alleles randomly drawn from genomic alleles (solid black curve) and disease-susceptible risk alleles (dashed grey curve) that share the same allele frequencies with T2D risk alleles in the European populations. Two-side p values were calculated by comparing dotted vertical lines against the null distributions of frequency-matched genomic alleles and disease-susceptible risk alleles. SNPs used in each figure are summarized in Table S4. (PDF) [file pgen.1002621.s004.pdf]

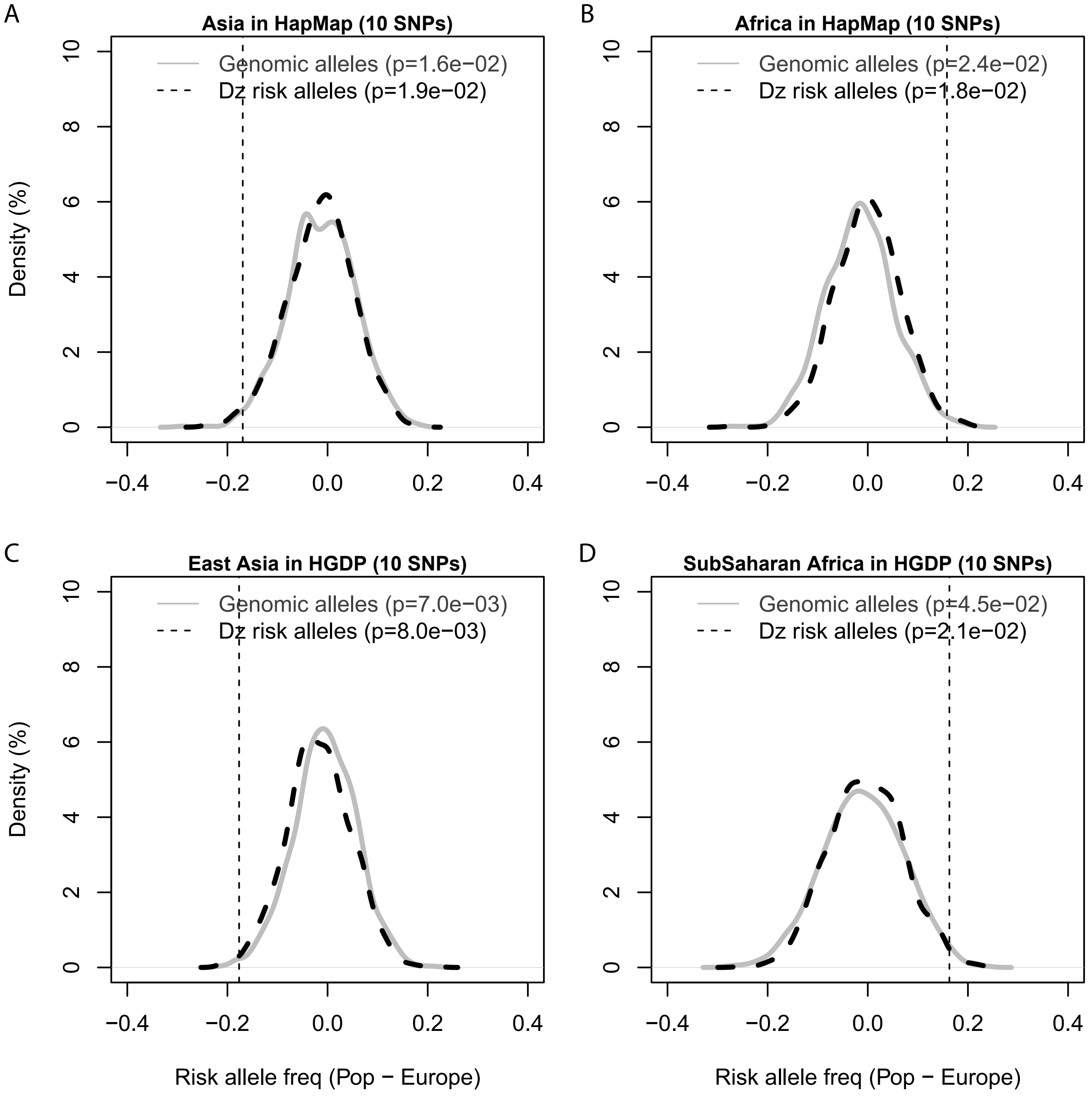

A
Asia in HapMap (10 SNPs)
Genomic alleles (p=1.6e−02)
Dz risk alleles (p=1.9e−02)
B
Africa in HapMap (10 SNPs)
Genomic alleles (p=2.4e−02)
Dz risk alleles (p=1.8e−02)
C
East Asia in HGDP (10 SNPs)
Genomic alleles (p=7.0e−03)
Dz risk alleles (p=8.0e−03)
D
SubSaharan Africa in HGDP (10 SNPs)
Genomic alleles (p=4.5e−02)
Dz risk alleles (p=2.1e−02)
Density (%)
0
2
4
6
8
10
−0.4
−0.2
0.0
0.2
0.4
Risk allele freq (Pop − Europe)
