# Supplementary material for: Type 2 Diabetes Risk Alleles Demonstrate Extreme Directional Differentiation among Human Populations, Compared to Other Diseases
Source: PLoS Genet. 2012 Apr 12;8(4):e1002621. doi: 10.1371/journal.pgen.1002621 (PMC3325177; doi:10.1371/journal.pgen.1002621)
Supplement: Figure S7 — RAF of Obesity risk alleles across the 11 HapMap populations. The frequencies of 11 independent multiethnic Obesity risk alleles are shown as heights of bars across the 11 HapMap populations. RAFs in the Asian populations (JPT, CHB, CHD) are shaded and listed on the left. RAFs in the African populations (ASW, MKK, LWK, YRI) are colored in grey and listed on the right. Higher RAFs in the African and lower RAFs in the Asian populations are only observed in 2 SNPs in FTO and 2 SNPs outside the gene region, including rs29941 and rs925946. Two SNPs in FTO are shared genetic risk variants with T2D. No consistent patterns are observed across obesity-specific risk alleles. The (anc) in the sub-title indicates that the risk allele is the ancestral allele from mammalian data, retrieved from the dbSNP. (PDF) [file pgen.1002621.s007.pdf]

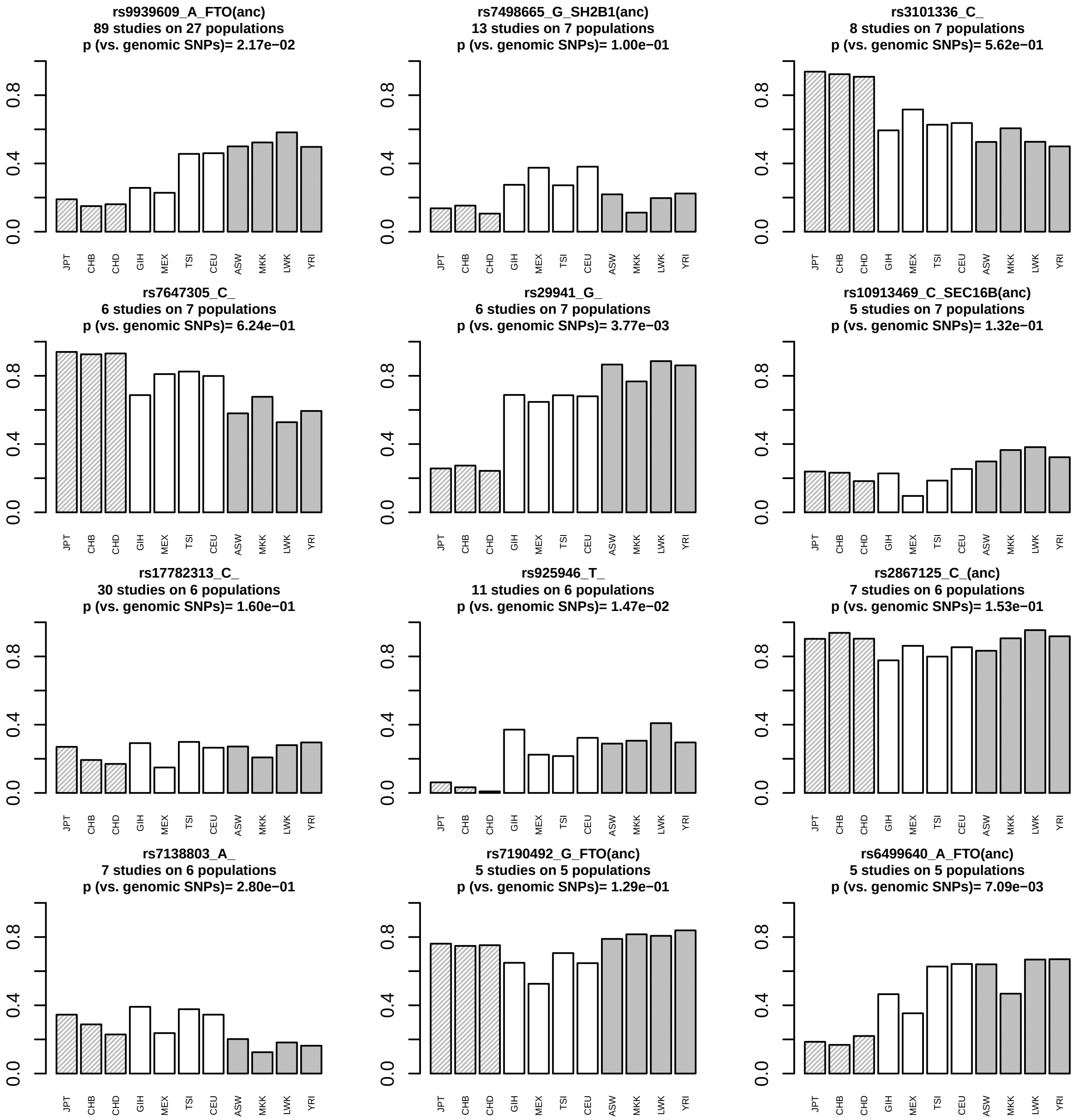
rs9939609_A_FTO(anc)
89 studies on 27 populations
p (vs. genomic SNPs)= 2.17e−02
rs7498665_G_SH2B1(anc)
13 studies on 7 populations
p (vs. genomic SNPs)= 1.00e−01
rs3101336_C_
8 studies on 7 populations
p (vs. genomic SNPs)= 5.62e−01
rs7647305_C_
6 studies on 7 populations
p (vs. genomic SNPs)= 6.24e−01
rs29941_G_
6 studies on 7 populations
p (vs. genomic SNPs)= 3.77e−03
rs10913469_C_SEC16B(anc)
5 studies on 7 populations
p (vs. genomic SNPs)= 1.32e−01
rs17782313_C_
30 studies on 6 populations
p (vs. genomic SNPs)= 1.60e−01
rs925946_T_
11 studies on 6 populations
p (vs. genomic SNPs)= 1.47e−02
rs2867125_C_(anc)
7 studies on 6 populations
p (vs. genomic SNPs)= 1.53e−01
rs7138803_A_
7 studies on 6 populations
p (vs. genomic SNPs)= 2.80e−01
rs7190492_G_FTO(anc)
5 studies on 5 populations
p (vs. genomic SNPs)= 1.29e−01
rs6499640_A_FTO(anc)
5 studies on 5 populations
p (vs. genomic SNPs)= 7.09e−03
0.0
0.4
0.8
JPT
CHB
CHD
GIH
MEX
TSI
CEU
ASW
MKK
LWK
YRI
