# Supplementary material for: Type 2 Diabetes Risk Alleles Demonstrate Extreme Directional Differentiation among Human Populations, Compared to Other Diseases
Source: PLoS Genet. 2012 Apr 12;8(4):e1002621. doi: 10.1371/journal.pgen.1002621 (PMC3325177; doi:10.1371/journal.pgen.1002621)
Supplement: Table S1 — Significance of T2D association and replication of the 12 cross-ethnic SNPs. (PDF) [file pgen.1002621.s008.pdf]

Table S1: Significance of T2D association and replication of the 12 cross-ethnic SNPs

SNP	Min(P value)	Number of populations with $p<5\times10^{-8}$
rs7903146	5.4×10^{-140}	13
rs10811661	7.8×10^{-15}	6
rs13266634	1.8×10^{-14}	3
rs4402960	8.6×10^{-16}	3
rs7754840	4.1×10^{-11}	5
rs5219	6.7×10^{-11}	3
rs1111875	9.1×10^{-15}	4
rs11196205	4.6×10^{-8}	1
rs8050136	1.7×10^{-17}	2
rs2237892	1.7×10^{-42}	4
rs7756992	1.9×10^{-12}	3
rs2074196	8.6×10^{-34}	4
