# Supplementary material for: Type 2 Diabetes Risk Alleles Demonstrate Extreme Directional Differentiation among Human Populations, Compared to Other Diseases
Source: PLoS Genet. 2012 Apr 12;8(4):e1002621. doi: 10.1371/journal.pgen.1002621 (PMC3325177; doi:10.1371/journal.pgen.1002621)
Supplement: Table S2 — Associations of 12 independent cross-ethnic T2D SNPs across 34 populations. (PDF) [file pgen.1002621.s009.pdf]

Table S2: Associations of 12 independent cross-ethnic T2D SNPs across 34 populations

dbSNP	Population	P value	Odds Ratio	PubMed
rs7903146 T vs. C	African_American	1.70E-08	1.45	17601994, 18443202
	African	0.00021	1.45	17206141
	American	1.00E-48	1.58	17003358, 16415884, 17463246
	Amish	0.008	1.57	16936218
	Arab	0.002	1.25	19368707
	Austrian	3.00E-07	1.52	17476472
	Brazilian	0.0025	1.57	19055834
	British	1.30E-11	1.36	16936215
	Caucasian_mixed	2.21E-51	1.44	19056611, 18694974, 18498634, 17977958, 16415884, 17725629, 16936217, 17020404, 18310307, 20581827, 16855264
	Danish	6.46E-13	1.48	16415884, 17206141
	Dutch	4.00E-05	1.41	17031610
	European_mixed	2.20E-51	1.51	18239663, 19734900, 17665514, 20081858, 19002430, 19149908, 17463248, 17668382, 17463246
	Finnish	2.30E-31	1.41	17463248, 17003358, 17671651, 17463246, 16936217
	French	2.10E-38	1.77	17293876, 17003360
	German	3.00E-05	1.36	17226113, 18546086
	Icelandic	5.19E-15	1.49	16415884, 17206141
	Indian	6.60E-07	1.40	17697858, 20694148, 17093941, 17665514
	Japanese	7.60E-12	1.57	17653210, 19012045, 17340123, 19401414, 20818381, 17245589
	Jewish	0.0001	1.27	18516622
	Meta_analysis	5.40E-140	1.47	17476472, 18445358, 19228405, 16855264
	Mexican	0.004	1.09	17259383
	Moroccan	2.90E-06	1.56	17476472
	Norwegian	1.89E-11	1.51	18972257, 19833889
	Polish	1.04E-05	1.38	17003358
	Scandinavian	0.000469	1.48	17003358
	Scottish	1.40E-12	1.48	17429603
	Spanish	0.01	1.29	18712344
	Swedish	1.00E-05	1.65	17618413, 19053027, 17003358, 17671651
rs10811661 T vs. C	American	7.80E-15	1.2	17463246
	Asian_mixed	0.0001	1.29	18469204
	Austrian	0.002	1.49	18461161
	Caucasian_mixed	1.45E-10	1.25	19056611, 20581827, 18176561
	Chinese	2.76E-05	1.24	18633108, 18469204, 20862305

	Danish	8.50E-06	1.91	18984664, 18176561
	European_mixed	7.80E-15	1.20	18048406, 17463248, 19002430, 17463249, 17463246
	Finnish	5.40E-08	1.2	17463249, 17463248, 17463246
	French	3.50E-08	1.39	18461161, 18368387
	Icelandic	0.0001	1.26	18176561, 19578363
	Italian	0.02	1.27	20403154
	Japanese	2.20E-08	1.26	18477659, 17928989, 18162508, 19033397, 19401414
	Korean	2.00E-08	1.52	18469204, 18991055, 19002430
	Norwegian	0.004	1.2	18437351
rs13266634 C vs. T	American	5.30E-08	1.12	17463246
	Asian_mixed	0.01	1.13	18469204
	Austrian	0.01	1.32	18461161
	Caucasian_mixed	1.52E-08	1.19	18498634, 18694974, 19056611, 20581827
	Chinese	0.002	1.17	20862305, 18633108, 17460697, 18469204
	Danish	0.01		18324385
	European_mixed	1.50E-08	1.13	17463248, 17463246, 17460697, 17463249, 19002430, 20081858, 18324385
	Finnish	6.80E-05	1.18	17463246, 17463248, 17463249
	French	6.10E-08	1.25	17293876, 18461161
	Icelandic	0.0006	1.19	17460697
	Japanese	1.80E-14	1.27	19401414, 19033397, 18162508, 17928989
	Jewish	0.02	1.14	18516622
	Korean	0.003	1.27	18991055, 19002430, 18469204
	Norwegian	0.00039	1.2	18437351
rs4402960 T vs. G	American	8.90E-16	1.14	19933996, 17463246
	Asian_mixed	0.012	1.14	18469204
	Austrian	0.03	1.25	18461161
	Caucasian_mixed	0.0008	1.24	19056611, 18694974
	Chinese	0.00024	1.23	20862305, 18633108
	European_mixed	8.60E-16	1.13	17463249, 19002430, 17463246, 17463248
	Finnish	1.70E-09	1.21	17463246, 17463248, 17463249
	French	0.002	1.14	18461161
	Indian	0.027	1.37	18598350
	Japanese	1.00E-06	1.26	18477659, 18162508, 19401414, 19033397, 18259684
	Korean	0.001	1.21	19002430, 18469204
	Norwegian	5.61E-05	1.29	19833889
	Swiss	0.01	1.29	19139842
rs7754840 C vs. G	American	4.10E-11	1.12	17463246
	Asian_mixed	0.0001	1.28	18469204
	Caucasian_mixed	3.11E-15	1.25	18694974, 20581827

	Chinese	8.91E-07	1.39	18633108, 18469204
	European_mixed	4.10E-11	1.12	17463248, 19002430, 17463246
	Finnish	0.0024	1.20	17463246, 18285412, 17463248
	French	1.60E-06	1.23	18461161
	Japanese	1.70E-10	1.34	19033397, 18477659, 19401414
	Jewish	3.00E-05	1.30	18461161, 18516622
	Korean	5.00E-11	1.35	18991055, 18469204, 19002430
rs5219 T vs. C	African_American	0.045	1.45	17601994
	American	6.70E-11	1.14	17463246
	Arab	0.148	1.07	19368707
	British	0.0333	1.49	14551916
	Caucasian_mixed	1.30E-09	1.30	17977958, 19056611
	Chinese	0.001	1.4	20079163
	European_mixed	6.70E-11	1.15	17463248, 17463246
	Finnish	1.00E-07	1.16	17463246, 18678618, 17463248
	Japanese	0.00025	1.24	19401414, 18162508
rs1111875 C vs. T	American	5.70E-10	1.13	17463246, 19933996
	Asian_mixed	0.003	1.16	18469204
	Caucasian_mixed	9.10E-15	1.17	20581827
	Chinese	0.0004	1.64	18633108
	Dutch	0.003	1.68	18231124
	European_mixed	5.70E-10	1.13	17463246, 17463248, 19002430
	Finnish	0.00017	1.10	17463248, 17463246
	French	7.30E-06	1.19	17293876
	Japanese	6.70E-12	1.30	17971426, 19401414, 17928989, 18162508, 18477659
	Korean	0.00018	1.25	18991055, 18469204, 19002430
rs11196205 C vs. G	African_American	0.022	1.27	17601994
	American	2.50E-05	1.22	17003358, 16415884
	Amish	0.05	1.45	16936218
	Caucasian_mixed	4.60E-08	1.31	16415884, 16936217
	Danish	0.0039	1.38	16415884
	Finnish	0.03	1.15	16936217
	German	0.038	2.11	17609304
	Icelandic	9.70E-05	1.29	16415884
	Japanese	0.0085	1.37	19012045, 17340123, 17653210
	Meta_analysis		1.24	19228405
rs8050136 A vs. C	African_American	0.001		20142250
	American	0.001		20142250
	Caucasian_mixed	1.70E-17	1.27	19056611, 18694974
	Danish	1.60E-05	1.13	19079260
	European_mixed	7.30E-14	1.21	17463249, 19002430, 17463248

	Finnish	0.0063	1.15	17463248, 17463249
	Icelandic	1.60E-05	1.13	19079260
	Japanese	0.022	1.22	17928989
	Norwegian	0.027		19833889
	Pima_Indian	0.03	1.2	19008344
	Scottish	0.0143	1.11	18591388
rs2237892 C vs. T	Asian_mixed	2.00E-39	1.41	18711367
	Caucasian_mixed	0.0027	1.14	20581827
	Chinese	4.20E-09	1.46	19448982, 19308350, 18711367
	European_mixed	0.00072	1.4	18711367
	Japanese	1.70E-28	1.38	18711367, 20818381, 19401414
	Korean	1.00E-05	1.52	18711367, 18991055
	Meta_analysis	1.70E-42	1.4	18711367
rs7756992 G vs. A	Asian_mixed	0.0001	1.28s	18469204
	Chinese	2.90E-05	1.29	18469204, 17460697, 18633108
	Danish	5.40E-05	1.21	17460697
	European_mixed	7.70E-10	1.2	17460697, 19002430
	French	2.30E-09	1.3	18461161
	Icelandic	0.00021	1.23	17460697
	Japanese	1.93E-12	1.26	18477659, 17928989, 19033397, 18162508, 19401414, 20818381
	Jewish	0.02	1.24	18461161
	Korean	8.20E-06	1.30	18469204, 19002430
rs2074196 G vs. T	Asian_mixed	9.90E-32	1.35	18711367
	Chinese	9.80E-10	1.29	18711367, 19308350
	European_mixed	0.017	1.23	18711367
	Japanese	9.80E-21	1.34	18711367
	Korean	2.10E-05	1.39	18711367
	Meta_analysis	8.60E-34	1.35	18711367
