# Supplementary material for: Type 2 Diabetes Risk Alleles Demonstrate Extreme Directional Differentiation among Human Populations, Compared to Other Diseases
Source: PLoS Genet. 2012 Apr 12;8(4):e1002621. doi: 10.1371/journal.pgen.1002621 (PMC3325177; doi:10.1371/journal.pgen.1002621)
Supplement: Table S3 — Between-study heterogeneity analysis of allelic Odds Ratios on T2D risk alleles. (PDF) [file pgen.1002621.s010.pdf]

Table S3: Between-study heterogeneity analysis of allelic odds ratios on T2D risk alleles

SNP	Study counts	Cochran's Q	I^2
rs7903146	48	116.80	60%
rs10811661	22	37.56	44%
rs13266634	22	25.31	17%
rs4402960	19	21.68	17%
rs7754840	15	82.03	83%
rs5219	10	16.38	45%
rs1111875	14	34.32	62%
rs11196205	7	12.91	54%
rs8050136	9	7.94	0%
rs2237892	6	31.51	84%
rs7756992	15	9.49	0%
rs2074196	2	5.34	81%
