# Supplementary material for: Type 2 Diabetes Risk Alleles Demonstrate Extreme Directional Differentiation among Human Populations, Compared to Other Diseases
Source: PLoS Genet. 2012 Apr 12;8(4):e1002621. doi: 10.1371/journal.pgen.1002621 (PMC3325177; doi:10.1371/journal.pgen.1002621)
Supplement: Table S4 — Summary of disease-susceptible SNPs used in the figures. (PDF) [file pgen.1002621.s011.pdf]

Table S4: Summary of disease-susceptible SNPs used in the figures

Disease	Figures	SNPs
T2D	S1	rs5219, rs1111875, rs2074196, rs2237892, rs4402960, rs7754840, rs7756992, rs7903146, rs8050136, rs10811661, rs11196205, rs13266634
	1, S3, 3A, 3B, 6A	rs1111875, rs2237892, rs4402960, rs7754840, rs7756992, rs7903146, rs8050136, rs10811661, rs11196205, rs13266634
	2+S2	rs1111875, rs2074196, rs2237892, rs4402960, rs7754840, rs7756992, rs7903146, rs8050136, rs10811661, rs11196205, rs13266634
	3C, 3D, 4, 5	rs1111875, rs2074196, rs2237892, 4402960, rs7754840, rs7756992, rs7903146, rs8050136, rs10811661, rs11196205, rs13266634
	7A	rs564398, rs864745, rs1111875, rs1153188, rs1470579, rs1617640, rs2237892, rs2641348, rs4402960, rs4607103, rs7172432, rs7578597, rs7593730, rs7754840, rs7756992, rs7901695, rs7903146, rs7961581, rs8050136, rs9465871, rs9939609, rs10811661, rs10923931, rs11196205, rs12243326, rs12255372, rs13266634
	7B	rs7901695, rs7903146
	7C	rs2237892, rs4402960, rs5015480, rs7754840, rs7756992, rs7901695, rs9465871, rs10811661, rs13266634
	7D	rs1111875, rs2237892, rs3802177, rs4402960, rs5015480, rs7578597, rs7754840, rs7756992, rs7901695, rs8050136, rs10811661, rs13266634
	7E	rs4402960, rs4506565, rs7903146, rs12255372
	8	rs243021, rs340874, rs864745, rs896854, rs1111875, rs1470579, rs1531343, rs1552224, rs1801282, rs4607103, rs7578326, rs7578597, rs7754840, rs7903146, rs7961581, rs10811661, rs10923931, rs11634397, rs11708067, rs13266634

	9A	rs5219, rs564398, rs864745, rs1111875, rs1153188, rs1359790, rs1436955, rs1470579, rs1617640, rs2074196, rs2237892, rs2237895, rs2237897, rs2383208, rs2970847, rs3802177, rs4402960, rs4607103, rs4712524, rs5015480, rs6769511, rs7172432, rs7578597, rs7593730, rs7754840, rs7756992, rs7895340, rs7901695, rs7903146, rs7961581, rs8050136, rs9295475, rs9300039, rs9465871, rs9939609, rs10010131, rs10811661, rs10906115, rs10923931, rs11196205, rs12243326, rs12255372, rs12779790, rs13266634, rs17036101
	9B	rs564398, rs864745, rs1111875, rs1153188, rs1359790, rs1436955, rs1470579, rs1617640, rs2237892, rs2383208, rs2641348, rs2970847, rs3802177, rs4402960, rs4607103, rs5015480, rs6769511, rs7172432, rs7578597, rs7593730, rs7754840, rs7756992, rs7901695, rs7903146, rs7961581, rs8050136, rs9295475, rs9465871, rs9939609, rs10811661, rs10906115, rs10923931, rs11196205, rs12243326, rs12255372, rs13266634
	S4, S5, S6	rs864745, rs1111875, rs2237892, rs4402960, rs7754840, rs7903146, rs8050136, rs10811661, rs11196205, rs13266634
Breast cancer	4,5	rs1219648, rs3803662, rs3817198, rs4415084, rs10941679, rs13387042
	S5, S6	rs889312, rs1219648, rs3803662, rs4973768, rs13387042
Celiac disease	4, 5	rs842647, rs917997, rs1464510, rs1738074, rs2327832, rs2816316, rs3184504, rs6441961, rs6822844, rs9811792, rs13151961, rs17810546
	S5, S6	rs653178, rs917997, rs1464510, rs1893217, rs2327832, rs2816316, rs6822844, rs11221332, rs11734090, rs12642902, rs13098911, rs13151961, rs17810546
Crohn's disease	4, 5	rs181359, rs212388, rs736289, rs1004819, rs1250550, rs1343151, rs2066844, rs2076756, rs2241880, rs2476601, rs2542151, rs2838519, rs2872507, rs3024505, rs3197999, rs3764147, rs4809330, rs6478108, rs6556412, rs6908425, rs7517847, rs7554511, rs10489629, rs10758669, rs10761659, rs10883365, rs10889677, rs11209026, rs12720356, rs17293632
	S5, S6	rs181359, rs1250550, rs1343151, rs2188962, rs2201841, rs2241880, rs2476601, rs2542151, rs2838519, rs2872507, rs3024505, rs3197999, rs3764147, rs3810936, rs6556412, rs6908425, rs7517847, rs10758669, rs10761659, rs11209026, rs12720356, rs17293632
Multiple sclerosis	4, 5	rs2104286, rs3135388, rs6498169, rs6897932, rs12044852, rs12722489
	S5, S6	rs2076530, rs3135338, rs3135388, rs6897932, rs7382297, rs9267954, rs12722489

Obesity	4, 5	rs6235, rs29941, rs925946, rs2867125, rs3101336, rs6499640, rs7138803, rs7190492, rs7498665, rs7647305, rs9939609, rs10913469, rs17782313
	S5, S6	rs29941, rs987237, rs2867125, rs7138803, rs7190492, rs7498665, rs8044769, rs9939609, rs10938397, rs17782313
	S7	rs29941, rs925946, rs2867125, rs3101336, rs6499640, rs7138803, rs7190492, rs7498665, rs7647305, rs9939609, rs10913469, rs17782313
Prostate cancer	4, 5, 6E	rs445114, rs1016343, rs1447295, rs1859962, rs4430796, rs5945619, rs6465657, rs6983267, rs7931342, rs9364554, rs10993994, rs16901979
	S5, S6	rs620861, rs1016343, rs1447295, rs1512268, rs2735839, rs4430796, rs5945619, rs6983267, rs7931342, rs10808556, rs10993994, rs13252298, rs16901979
Psoriasis	4, 5	rs20541, rs610604, rs2082412, rs2201841, rs4112788, rs12191877, rs17728338
	S5, S6	rs610604, rs1265181, rs2082412, rs2201841, rs4112788, rs6887695, rs10484554, rs17728338
Rheumatoid arthritis	4, 5	rs2476601, rs3761847, rs4750316, rs4810485, rs6822844, rs6920220, rs7574865, rs10499194
	S5, S6	rs1953126, rs2076530, rs2476601, rs3087243, rs3135363, rs4750316, rs4810485, rs6457617, rs6822844, rs6859219, rs6920220, rs7574865
Systemic lupus erythematosus (SLE)	4, 5	rs130073, rs416352, rs419132, rs707929, rs729302, rs805301, rs1264419, rs1265087, rs2187668, rs2227139, rs2395153, rs2395175, rs2476601, rs3024866, rs3024877, rs3024896, rs3130299, rs3131379, rs3132571, rs7574865, rs9888739, rs10484565, rs13277113
	S5, S6	rs2187668, rs2205960, rs2248932, rs2431697, rs3131379, rs4548893, rs4963128, rs6445975, rs6568431, rs7574865, rs9888739, rs10516487, rs12537284, rs13277113
T1D	4, 5, 6D	rs763361, rs1445898, rs1990760, rs2104286, rs2292239, rs2358994, rs2476601, rs2542151, rs3087243, rs3184504, rs3764021, rs7529353, rs12251307, rs12708716, rs17696376
	S5, S6	rs763361, rs1893217, rs1990760, rs2104286, rs2292239, rs2476601, rs3087243, rs3184504, rs9272346, rs11594656, rs12708716, rs13330041, rs17696736
Ulcerative colitis	4, 5	rs1558744, rs1801274, rs2201841, rs2395185, rs2836878, rs3024505, rs3806308, rs6426833, rs10758669, rs11209026, rs17085007
	S5, S6	rs1558744, rs1801274, rs2395185, rs2836878, rs3024505, rs3806308, rs6017342, rs6426833, rs11209026
Colorectal cancer	6B	rs6983267, rs10505477

Cleft palate	6C	rs742071, rs8069536, rs17352100
Parkinson's disease	6F	rs356220, rs393152, rs2736990, rs3857059, rs17563986
Lung cancer	6G	rs401681, rs1051730, rs2736100, rs16969968
Melanoma	9C	rs258322, 401681, rs1126809, rs1335510, rs1393350, rs1805007, rs1805008, rs1847142, rs2218220, rs2284063, rs2353033, rs4238833, rs4778138, rs4785763, rs7023329, rs7188458, rs8059973, rs10757257, rs12918773, rs16891982
	9D	rs258322, rs401681, rs1335510, rs1393350, rs1805007, rs2218220, rs2284063, rs2353033, rs4238833, rs4778138, rs4785763, rs6001027, rs7023329, rs7188458, rs8059973, rs10757257, rs12918773
