# Supplementary material for: Type 2 Diabetes Risk Alleles Demonstrate Extreme Directional Differentiation among Human Populations, Compared to Other Diseases
Source: PLoS Genet. 2012 Apr 12;8(4):e1002621. doi: 10.1371/journal.pgen.1002621 (PMC3325177; doi:10.1371/journal.pgen.1002621)
Supplement: Table S5 — Statistical significance of ethnic disparity of PGR across 11 HapMap3 populations on 40 diseases. (PDF) [file pgen.1002621.s012.pdf]

Table S5: Statistical significance of ethnic disparity of PGR across 11 HapMap3 populations on 40 diseases

disease	p_Asi	p_Eur	p_Afr	SNPs
Type 2 diabetes	3.0E-05	0.81	4.7E-03	10
Colorectal cancer	1.3E-03	0.03	5.7E-04	2
Cleft palate	1.4E-02	0.78	0.16	3
Schizophrenia	0.11	0.44	0.03	1
Crohn's disease	0.12	0.74	0.29	20
Atrial fibrillation	0.13	0.32	0.70	1
Glioma	0.14	0.07	0.97	14
Multiple sclerosis	0.14	0.35	0.58	5
Bladder cancer	0.17	0.60	0.72	8
Osteoarthritis	0.18	0.34	0.35	1
Migraine	0.21	0.34	0.65	1
Restless legs syndrome	0.30	0.23	0.91	6
Pseudoexfoliation syndrome	0.38	0.21	0.83	1
Exfoliation glaucoma	0.38	0.21	0.83	1
Type 1 diabetes	0.38	5.0E-03	2.6E-02	15
Age related macular degeneration	0.40	0.84	0.45	2
Psoriatic arthritis	0.41	0.13	0.90	4
Follicular lymphoma	0.42	0.59	0.59	3
Systemic sclerosis	0.46	0.11	0.95	3
Systemic lupus erythematosus	0.46	0.56	0.77	23
Obesity	0.53	0.25	0.40	12
Myocardial infarction	0.53	0.48	0.37	1
Coronary artery disease	0.54	0.48	0.36	1
Rheumatoid arthritis	0.56	0.83	0.51	8
Prostate cancer	0.56	0.02	0.05	12
Amyotrophic lateral sclerosis	0.59	0.32	0.93	1
Breast cancer	0.60	0.43	0.37	6
Melanoma	0.61	0.94	0.51	1
Inflammatory bowel disease	0.61	0.52	0.92	2
Parkinson's disease	0.63	5.7E-04	0.99	5
Lung cancer	0.67	0.05	0.65	4
Psoriasis	0.72	0.53	0.89	7
Essential tremor	0.80	0.47	0.35	1
Alzheimer's disease	0.87	0.74	0.87	3
Intracranial aneurysm	0.91	0.15	0.45	5
Celiac disease	0.91	0.40	0.69	11

Ulcerative colitis	0.92	0.68	0.94	11
Asthma	0.93	0.15	0.37	1
Chronic lymphocytic leukemia	0.95	0.12	0.22	1
Hodgkin's lymphoma	0.98	0.99	0.90	1
